# Chiral Polymers from Norbornenes Based on Renewable Chemical Feedstocks

**DOI:** 10.3390/polym14245453

**Published:** 2022-12-13

**Authors:** Ivan V. Nazarov, Danil P. Zarezin, Ivan A. Solomatov, Anastasya A. Danshina, Yulia V. Nelyubina, Igor R. Ilyasov, Maxim V. Bermeshev

**Affiliations:** 1 A.V. Topchiev Institute of Petrochemical Synthesis, RAS, 29 Leninskiy Pr., 119991 Moscow, Russia; 2 A.N. Nesmeyanov Institute of Organoelement Compounds, Russian Academy of Sciences, Vavilov Street 28, 119991 Moscow, Russia; 3 Moscow Institute of Physics and Technology, National Research University, Institutskiy Per., 9, 141700 Dolgoprudny, Russia; 4 Nelubin Institute of Pharmacy, Sechenov First Moscow State Medical University, Trubetskaya Str. 8/2, 119991 Moscow, Russia

**Keywords:** chiral polymer, renewable feedstock, pinene, menthol, borneol, norbornene, polynorbornene, ring-opening metathesis polymerization

## Abstract

Optically active polymers are of great interest as materials for dense enantioselective membranes, as well as chiral stationary phases for gas and liquid chromatography. Combining the versatility of norbornene chemistry and the advantages of chiral natural terpenes in one molecule will open up a facile route toward the synthesis of diverse optically active polymers. Herein, we prepared a set of new chiral monomers from *cis*-5-norbornene-2,3-dicarboxylic anhydride and chiral alcohols of various natures. Alcohols based on cyclic terpenes ((-)-menthol, (-)-borneol and pinanol), as well as commercially available alcohols (S-(-)-2-methylbutanol-1, S-(+)-3-octanol), were used. All the synthesized monomers were successfully involved in ring-opening metathesis polymerization, affording polymers in high yields (up to 96%) and with molecular weights in the range of 1.9 × 10^5^–5.8 × 10^5^ (M_w_). The properties of the metathesis polymers obtained were studied by TGA and DSC analysis, WAXD, and circular dichroism spectroscopy. The polymers exhibited high thermal stability and good film-forming properties. Glass transition temperatures for the prepared polymers varied from −30 °C to +139 °C and, therefore, the state of the polymers changed from rubbery to glassy. The prepared polymers represent a new attractive platform of chiral polymeric materials for enantioselective membrane separation and chiral stationary phases for chromatography.

## 1. Introduction

Synthetic optically active polymers are of great interest both in academic and industrial fields. On the one hand, such polymers are widely studied and used in asymmetric catalysis [1,2,3] and as materials for dense enantioselective membranes [4,5,6], chiral stationary phases for gas and liquid chromatography [7,8,9], and also for mimicking biological properties [10,11,12]. On the other hand, these polymers are suitable objects for systematic structure–property study. There are three main approaches to achieving the optical activity of polymers. The first way is the formation of chiral centers in main chains [13,14]. The second one is the introduction of chiral moieties in side chains of polymers [15,16] and in the case of the third way, optical activity appears due to a supramolecular structure [17,18].

**Figure 1:**
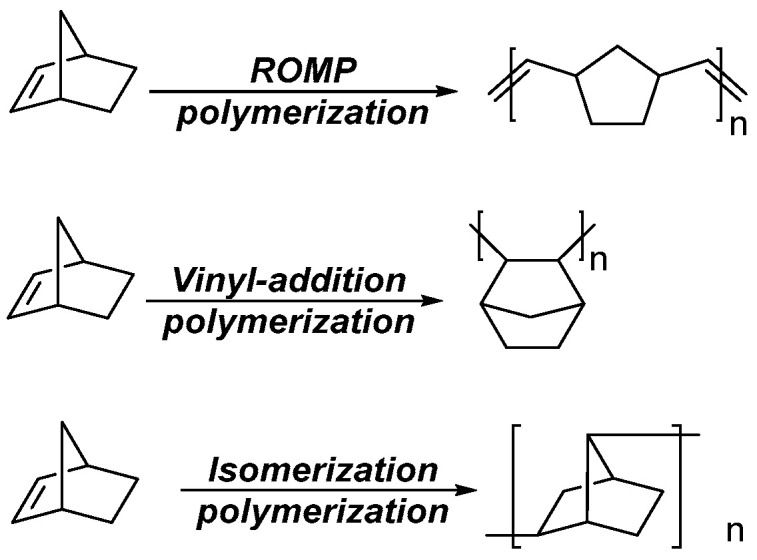
The ways of norbornene-type monomers polymerization.

Metathesis polymerization of various monomers is a powerful tool for obtaining all three types of polymers. At the same time, ring-opening metathesis polymerization (ROMP) of functionalized norbornenes is the most versatile methodology in the design and preparation of various polymers with the required architecture [19,20,21]. There are several significant benefits of this tool. Norbornene-type monomers can be readily prepared by using well-known cycloaddition (e.g., the Diels–Alder and [2 + 2 + 2]-cycloaddition reactions) and other reactions [22,23,24,25], and these monomers show high reactivity in ROMP polymerization. Moreover, such monomers can be polymerized according to three different mechanisms, affording polymers with different structures of main chains (Figure 1) [19,26,27,28,29]. In turn, well-defined Grubbs catalysts exhibit good tolerance towards various functional groups and allow the control of tacticity, configuration of backbones, the composition of copolymers, molecular weights, and molecular weight distributions.

Some chiral polymers from optically pure isomers of norbornene derivatives were earlier described. The most investigated among these monomers are N-substituted norbornene-2,3-dicarboximides with a chiral group at nitrogen atom [15,30] and norbornenes bearing ester [31,32,33] or ether [34,35] moiety between norbornene skeleton and a chiral fragment. At the same time, monoterpenes, such as α- and β-pinenes, borneol, and menthol are attractive synthetic building blocks and the source of available chiral moieties that are obtained from renewable feedstock. They are widely represented in nature. Therefore, it seems an attractive strategy to combine a norbornene moiety as a polymerizable part with a fragment of a terpene as the source of chirality in the same molecule to develop new optically active polymers for the structure–property study.

Here we report the preparation and ROMP polymerization of a new set of norbornene-type monomers based on alcohols from cyclic terpenes ((-)-menthol, (-)-borneol, and pinanol) as well as commercially available alcohols (S-(-)-2-methylbutanol-1, S-(+)-3-octanol). As a result, a series of high-molecular-weight polymers with good film-forming properties was successfully obtained. The prepared polymers represent a new attractive platform of chiral polymeric materials for enantioselective membrane separation and chiral stationary phases for chromatography. The properties of the metathesis polymers obtained were studied by TGA and DSC analysis, WAXD, and circular dichroism spectroscopy.

## 2. Materials and Methods

### 2.1. Materials

(-)-Menthol, (-)-borneol, S-(+)-3-octanol, S-(-)-2-methylbutanol-1, the 2nd generation Grubbs catalysts, dicyclopentadiene, 1,2,4-trichlorbenzene, maleic anhydride, Et_3_N, SOCl_2_, and *cis*-5-norbornene-*endo*-2,3-dicarboxylic anhydride (*endo*-NDA) were purchased from commercial suppliers (Sigma-Aldrich, ABCR GmbH, and TCI) and used as received unless otherwise noted. Methylene chloride and 1,2-dichloroethane were distilled over CaH_2_ under argon atmosphere and stored over molecular sieves (4 Å). Toluene was distilled in argon atmosphere and stored over molecular sieves (4 Å). ((1R,2S,5R)-6,6-Dimethylbicyclo[3.1.1]heptan-2-yl)methanol (pinanol) was prepared according to the procedure described earlier [36].

### 2.2. Physico-Chemical Characterization

NMR spectra were recorded on a Bruker Ascend 400 spectrometer at 400.1 MHz (^1^H), 100.6 MHz (^13^C) and on a Bruker MSL-300 spectrometer at 300 MHz (^1^H). Chemical shifts (δ) are reported in parts per million (ppm) relative to the reference (residual CHCl_3_ signal) for ^1^H and ^13^C NMR spectra. Each sample of a polymer for NMR analysis was dissolved in CDCl_3_ up to a concentration of 10%.

Gel-permeation chromatography (GPC) analysis of the polymers was performed on Agilent 1280 Infinity II system with a differential refractometer (THF as the eluent, flow rate 0.3 mL/min). Molecular mass and polydispersity were calculated by standard procedure relative to monodispersed polystyrene standards.

Calorimetric measurements were conducted using a “Mettler” TA-4000 differential scanning calorimeter (Mettler Toledo, Giesen, Germany) at a heating rate of 20 °C/min under argon. TGA measurements were carried out on “TGA/DSC 1” (Mettler Toledo, Polaris Parkway, OH, USA) in argon and in air at the heating rate of 10 °C/min from 30 to 1000 °C.

Wide-angle X-ray diffraction (WAXD) data were obtained using a two-coordinate AXS detector (Bruker, Bremen, Germany) and Cu Kα emission (wavelength of 0.154 nm).

Specific rotation was measured using KRÜSS P3000 polarimeter in CHCl_3_ or THF (HPLC grade). Circular dichroism (CD) spectra of the monomers and polymers were measured in chloroform or THF at the concentration of 1 mg/mL. Spectra were recorded with spectropolarimeter J810 (Jasco, Hachioji city, Tokio, Japan) in the 220–450 nm range (50 nm/min, 1 nm slit width) in 0.1 cm path-length quartz cells with a detachable window (Hellma, Jena, Germany). The baseline spectrum was recorded from pure chloroform or THF.

The density of polymer films was calculated by the hydrostatic weighing method according to the following procedure. A sample of the film was weighed on analytical balance (m_dry_). Thin copper wire was hung on a beam over the balance. The sample was fixed on the lower end of the wire, immersed in the beaker with methanol placed on the balance, and weighed (m_1_). Then, the sample was withdrawn and the free wire end immersed in methanol was weighed (m_2_). The density of the film sample was calculated by the formula: d = d_S_ m_dry_/(m_dry_ – (m_1_ – m_2_)), where d_S_ = 0.791 g/mL is the density of methanol.

X-ray diffraction data for **M2** were collected at 120 K with a Bruker APEXII DUO CCD diffractometer, using graphite monochromated Cu-Kα radiation (λ = 1.54178 Å, ω-scans), while those for **M1**, at 100 K with a Bruker Quest D8 CMOS diffractometer, using graphite monochromated Mo-Kα radiation (λ = 0.71073 Å, ω-scans). Structures were solved using Intrinsic Phasing with the ShelXT [37] structure solution program in Olex2 [38] and then refined with the XL [39] refinement package using Least-Squares minimization against F^2^ in the anisotropic approximation for non-hydrogen atoms. Positions of hydrogen atoms were calculated, and they were refined in the isotropic approximation within the riding model. Crystal data and structure refinement parameters are given in Table S1. CCDC 2213611 (**M2**) and 2213612 (**M1**) contain supplementary crystallographic information for this paper.

High-resolution mass spectra were recorded on Bruker maXis QTOF (tandem quarrupole/time-of-flight mass analyzer) mass spectrometers equipped with an ESI source. The m/z scanning range was 50–1600. External calibration of the mass scale was carried out using a low-concentration calibration solution “Tuning mix” (Agilent Technologies, Santa Clara, California, USA). Samples were injected using a 500 μL Hamilton RN 1750 syringe (Hamilton, Biel, Switzerland). The measurements were carried out in the positive ion mode (+) (grounded spray needle, −4500 V high-voltage capillary; HV End Plate Offset: −500 V). Nitrogen was used as a nebulizer gas (1.0 bar) and dry gas (4.0 L/min, 200 °C). The data were processed using the Bruker Data Analysis 4.0 software.

### 2.3. Film Preparation

The polymer films were prepared by casting from 5 wt.% chloroform solution of a polymer. The solution was poured into a steel cylinder with a diameter of 7 cm and a stretched cellophane bottom. The solvent was allowed to evaporate slowly at room temperature to yield the desired polymer film. After the formation of the films, the cellophane was detached, and the films were dried under vacuum at room temperature to a constant weight. A thermal treatment was not applied. The thickness of the films formed was in the range of 40–60 µm.

### 2.4. Synthetic Part

*Cis*-5-norbornene-*exo*-2,3-dicarboxylic anhydride (*exo*-NDA). In a 1000 mL two-necked round bottom flask fitted with reflux, dropping funnel with dicyclopentadiene (DCPD, 122.4 mL, 0.909 mol) and a magnetic stirring bar, was placed 300 mL of 1,2,4-trichlorbenzene and 178 g of maleic anhydride (1.818 mol). The solution was heated to 200 °C and DCPD added dropwise over 20 min. The solution acquired a yellow color, which changed to dark brown upon further heating. The resulting solution was stirred for 4 h at 200 °C. After that, it was cooled down and left overnight after the addition of *n*-hexane. The next day, the crystal precipitate was formed in the flask. The precipitate was separated and recrystallized 4 times from the minimum amount of benzene. The white crystalline substance was obtained (54.6 g, yield 18%). M.p.—145–149 °C

^1^H NMR (300 MHz, CDCl_3,_ δ, ppm): 1.50 (dd, 2H, RCH_2_, J = 10.20); 2.97 (s, 2H); 3.42 (s, 2H); 6.30 (s, 2H, R-CH=CH-R).
^13^C NMR (100 MHz, CDCl3, δ, ppm): 44.07; 46.82; 48.71; 137.91; 171.59.

### 2.5. General Procedure for the Synthesis of Monomers ***M1**–**M3***

The synthesis of **M1**. The first step was performed similarly to the previously described procedure with minor differences [40]. A 250 mL round-bottom flask was equipped with a magnetic stirring bar, and then was filled with 13.4 g (82 mmol) of *exo*-NDA, 200 mL of dry toluene, 29.5 mL of triethylamine (210 mmol) and 57.56 g (370 mmol) of (-)-menthol. The mixture was stirred for 96 h (24 for borneol ester) at 55 °C with the conversion control by ^1^H NMR spectroscopy. The remaining solvent and triethylamine were evaporated at a rotary evaporator. The resulting residue was redissolved in 125 mL of diethyl ether. The solution was washed with 10% hydrochloric acid (3 × 120 mL). The aqueous layer was extracted with diethyl ether (5 × 200 mL). The organic layers were combined and extracted with saturated K_2_CO_3_ solution (4 × 250 mL). The resulting aqueous phase was washed once with diethyl ether (150 mL). Then, the aqueous phase was acidified with hydrochloric acid and extracted with methylene chloride (5 × 200 mL). The combined organic layers were dried with anhydrous magnesium sulfate filtered and the remaining solvents were evaporated on a rotary evaporator. As a result, 26.24 g (96% yield) of crude (-)-menthyl ester of *exo*-norbornene dicarboxylic acid was obtained.

The second and third steps. In a 100 mL round-bottom flask equipped with a magnetic stirring bar, 11.34 g (35 mmol) of (-)-menthyl *exo*-norbornene dicarboxylic acid monoester, 25.5 mL (351 mmol) of thionyl chloride, and 80 mL of absolute toluene were placed with a couple of drops of DMF. The mixture was stirred for 4 h at 90 °C. The remaining excess of thionyl chloride was removed under vacuum, and the resulting product was dissolved in 150 mL of dry toluene and 3.66 mL of pyridine (1.3 eq.). (-)-Menthol (4.66 g, 30 mmol) was added to the prepared solution. The resulting mixture was heated to 90 °C and stirred for two hours. Then, toluene was evaporated on a rotary evaporator. The resulting product was recrystallized from ethanol. The yield of **M1** was 13.82 g (86% at this stage). The crystals of **M1** suitable for X-ray analysis were prepared by the slow evaporation of methanol solution of **M1**. [α]_D_^25^ = −75 deg·mL·g^−1^·dm^−1^ in CHCl_3_ (C = 1). ESI-(+)HRMS, m/z: 459.3472, calcd for C_29_H_47_O_4_: 459.3469 [M + H]^+^.

^1^H NMR (400 MHz, CDCl_3_, δ, ppm): 6.26–6.24 (m, 2H, R-HC=CH-R), 4.61–4.76 (m, 2H), 3.15–3.14 (m, 1H), 3.05–3.04 (m, 1H), 2.63–2.62 (m, 1H), 2.55–2.54 (m, 1H), 2.13–2.04 (m, 3H), 2.03–1.86 (m, 2H), 1.76–1.60 (m, 4H), 1.56–1.30 (m, 5H), 1.62–0.76 (m, 24H).
^13^C NMR (100 MHz, CDCl_3_, δ, ppm): 172.93–172.78, 138.15–137.77, 74.52–74.24, 47.53, 47.34, 46.95, 46.88, 46.78, 45.68, 45.19, 40.68, 40.23, 34.33, 31.49–31.35, 26.19, 25.96, 23.42, 23.35, 22.07, 20.95, 16.57, 16.25.
Monomer **M2**

*Endo*-NDA was used instead of *exo*-NDA for **M2** synthesis. The yield of **M2** was 76%. The crystals of **M2** suitable for X-ray analysis were prepared by the slow evaporation of the methanol solution of **M2**. [α]_D_^20^ = −125 deg·mL·g^−1^·dm^−1^ in CHCl_3_ (C = 1). ESI-(+)HRMS, m/z: 459.3461, calcd for C_29_H_47_O_4_: 459.3469 [M + H]^+^.

^1^H NMR (400 MHz, CDCl_3_, δ, ppm): 6.28 (br. S., 1H), 6.20 (br.s., 1H), 4.68–4.53 (m, 2H), 3.25–3.15 (m, 4H), 2.05–1.96 (m, 2H), 1.95–1.82 (m, 2H), 1.68–1.62 (m, 4H), 1.47–1.23 (m, 7H), 1.10–0.96 (m, 2H), 0.94–0.79 (m, 17H), 0.78–0.70 (m, 6H).
^13^C NMR (100 MHz, CDCl_3_, δ, ppm): 171.98, 171.79, 135.32, 134.52, 74.36, 74.00, 48.62, 48.18, 48.14, 47.48, 47.04, 46.78, 40.95, 40.84, 34.50, 34.45, 31.59, 31.45, 26.25, 25.96, 23.42, 23.37, 22.19, 21.09, 21.06, 16.46, 16.36.
Monomer **M3**

The yield of **M3** was 72%. [α]_D_^20^ = −42 deg·mL·g^−1^·dm^−1^ in CHCl_3_ (C = 1). ESI-(+)HRMS, m/z: 455.3156, calcd for C_29_H_43_O_4_: 455.3156 [M + H]^+^.

^1^H NMR (400 MHz, CDCl_3_, δ, ppm): 6.24 (br. S., 2H), 3.11–3.07 (m, 2H), 2.68–2.63 (m, 2H), 2.43–2.24 (m, 2H), 2.21–2.14 (m, 1H), 2.00–1.85 (m, 2H), 1.81–1.63 (m, 4H), 1.55–1.46 (m, 1H), 1.36–1.12 (m, 5H), 1.12–0.96 (m, 2H), 0.92–0.82 (m, 20H).
^13^C NMR (100 MHz, CDCl_3_, δ, ppm): 173.62, 173.61, 137.99, 137.94, 80.40, 80.15, 48.96, 48.58, 47.82, 47.70, 47.38, 45.91, 45.35, 44.86, 44.84, 36.87, 36.14, 28.03, 27.24, 27.22, 19.71, 18.85, 13.65, 13.51.

### 2.6. General Procedure for the Synthesis of Monomers ***M4**–**M6***

Synthesis of **M5**. A 50 mL one-neck flask was equipped with a reflux condenser, 0.93 g (0.57 mmol) *exo*-NDA, (*S*)-(-)-2-methylbutanol 1.1 g (1.25 mmol), 0.06 g (0.61 mmol) *para*-toluenesulfonic acid, and 30 mL toluene(abs) as a solvent. Molecular sieves (4 Å) were used as a drying agent. The reaction mixture was refluxed for 14 h and the conversion was monitored by ^1^H NMR spectroscopy. The mixture was cooled and washed with saturated solution of K_2_CO_3_ (2 × 15 mL). After that, the organic layer was washed with water (15 mL), brine (15 mL) and dried over MgSO_4_. Then, it was filtered and the toluene was evaporated on a rotary evaporator. The product was purified by silica gel column chromatography using hexanes/ethyl acetate (40/1) as an eluent. The product was obtained as a colorless viscous oil (1.3 g, 71% yield). [α]_D_^20^ = +7 deg·mL·g^−1^·dm^−1^ in THF (C = 1). ESI-(+)HRMS, m/z: 321.2038, calcd for C_29_H_29_O_4_: 321.2060 [M + H]^+^.

^1^H NMR (300 MHz, CDCl_3_, δ, ppm): 6.15 (br. s, 2H, R-CH=CH-R), 3.96–3.67 (M, 4H), 3.02 (s, 2H), 2.57 (s, 2H), 2.07 (m, 2H), 1.67–1.55 (m, 2H), 1.45–1.29 (m, 3H), 1.23–1.03 (m, 2H), 0.92–0.77 (m, 12H).
^13^C NMR (100 MHz, CDCl_3_, δ, ppm): 172.99, 137.36, 68.82, 46.77, 45.13, 44.80, 33.50, 33.46, 25.48, 15.87, 15.83, 10.66, 10.61.
Monomer **M4**

The monomer yield was 68%. [α]_D_^20^ = −11 deg·mL·g^−1^·dm^−1^ in CHCl_3_ (C = 1). ESI-(+)HRMS, m/z: 455.3153, calcd for C_29_H_43_O_4_: 455.3156 [M + H]^+^.

^1^H NMR (400 MHz, CDCl_3_, δ, ppm): 6.20 (s, 2H, R-CH=CH-R), 4.08–4.02 (m, 2H), 3.94–3.89 (m, 2H), 3.08–3.05 (m, 2H), 2.60–2.57 (m, 2H), 2.38–2.30 (m, 4H), 2.13–2.08 (m, 1H), 1.95–1.84 (m, 10H), 1.52–1.41 (m, 3H), 1.19–1.17 (m, 6H), 1.00–0.96 (m, 6H), 0.94–0.90 (m, 2H).
^13^C NMR (100 MHz, CDCl_3_, δ, ppm): 173.72, 173.67, 138.09, 138.07, 65.51, 69.30, 47.49, 47.47, 45.90, 45.82, 45.73, 45.54, 46.40, 43.18, 41.39, 41.37, 40.41, 40.30, 38.64, 33.12, 33.07, 27.99, 27.97, 25.98, 23.39, 23.34, 18.94, 18.81.
Monomer **M6**

The monomer yield was 54%. [α]_D_^20^ = +4 deg·mL·g^−1^·dm^−1^ in CHCl_3_ (C = 1). ESI-(+)HRMS, m/z: 407.3155, calcd for C_25_H_43_O_4_: 407.3156 [M + H]^+^.

^1^H NMR (400 MHz, CDCl_3_, δ, ppm): 6.17 (s, 2H, R-CH=CH-R), 4.74–4.71 (m, 2H), 3.03–3.02 (m, 2H), 2.54–2.53 (m, 2H), 2.11–2.09 (m, 1H), 1.52–1.42 (m, 9H), 1.37–1.17 (m, 12H), 0.87–0.83 (m, 12H).
^13^C NMR (100 MHz, CDCl_3_, δ, ppm): 173.15, 138.03, 137.99, 75.84, 75.79, 47.45, 47.37, 46.19, 46.08, 45.33, 33.21, 33.13, 31.92, 31.82, 26.55, 26.54, 25.05, 24.93, 22.62, 14.09, 14.06, 9.63, 9.50.
Epoxide based on **M5**
^1^H NMR (400 MHz, CDCl_3,_ δ, ppm): 4.03–3.72 (M, 4H), 3.18 (s, 2H), 2.82 (s, 2H), 2.78 (s, 2H), 1.76–0.84 (m, 20H).
^13^C NMR (100 MHz, CDCl_3,_ δ, ppm): 172.11, 69.68, 50.68, 47.08, 40.97, 34.02, 33.98, 26.03, 23.27, 16.43, 16.40, 11.23, 11.19.
MS (EI, m/z (intensity, %)): 338 (2%, M^+^), 181 (60%, C_9_H_9_O_4_^+^).

### 2.7. General Procedure of ROMP Polymerization (***MP1**–**MP6***)

The synthesis of **MP5**. The monomer **M5** (3.12 g, 9.7 mmol) was dissolved in 15.9 mL of absolute 1,2-dichloroethane and the solution of the 2-st generation Grubbs catalyst 3.52 mL (0.0129 mmol, 3.36 × 10^−3^ M) was added to the monomer solution at stirring. The stirring was continued for 2 h at 45 °C. The polymerization was terminated by the addition of ethyl vinyl ether with following stirring for 10 min. Then, the polymer solution was precipitated by methanol containing the inhibitor (2,2′-methylenebis(6-tert-butyl-4-methylphenol)). The polymer was separated, washed with several portions of methanol, and dried in vacuum. The polymer was twice reprecipitated by methanol from the chloroform solution and dried in vacuum to a constant weight (3.00 g, 96% yield). M_w_ = 4.57 × 10^5^, M_w_/M_n_ = 2.2. [α]_D_^22^ = 9 deg·mL·g^−1^·dm^−1^ in THF (C = 1).

^1^H NMR (400 MHz, CDCl_3_, δ, ppm): 5.51–5.23 (m, 2H, HRC=CRH), 3.94–0.72 (m, 31H).
^13^C NMR (100 MHz, CDCl_3_, δ, ppm): 178.14–178.10 (C=O), 133.37–131.80, 69.74–69.30, 53.91–52.62, 45.71–44.80, 41.25–39.45, 34.27–33.96, 26.31–26.05, 16.72–16.33, 11.41–11.23.
Polymer **MP1**

The polymer yield was 96%. M_w_ = 4.88 × 10^5^, M_w_/M_n_ = 2.1. [α]_D_^22^ = −59 deg·mL·g^−1^·dm^−1^ in CHCl_3_ (C = 1).

^1^H NMR (400 MHz, CDCl_3_, δ, ppm): 5.55–4.51 (m, 4H), 3.56–0.56 (m, 42H).
^13^C NMR (100 MHz, CDCl_3_, δ, ppm): 172.87–171.44, 134.54–131.65, 74.50–74.00, 54.31–52.07, 47.55–44.51, 41.26–39.18, 34.72–34.13, 31.90–31.32, 26.62–25.70, 23.86–23.18, 22.61–21.82, 24.41–20.85, 16.97–16.10.
Polymer **MP2**

The polymer yield was 74%. M_w_ = 1.9 × 10^5^, M_w_/M_n_ = 1.6. [α]_D_^22^ = −64 deg·mL·g^−1^·dm^−1^ in CHCl_3_ (C = 1).

^1^H NMR (400 MHz, CDCl_3_, δ, ppm): 5.74–5.28 (m, 2H), 4.81–4.55 (m, 2H), 3.31–0.45 (m, 42H).
^13^C NMR (100 MHz, CDCl_3_, δ, ppm): 171.94–170.91, 132.14–130.53, 75.12–73.81, 52.21–15.62.
Polymer **MP3**

The polymer yield was 94%. M_w_ = 4.69 × 10^5^, M_w_/M_n_ = 2.1. [α]_D_^22^ = −34 deg·mL·g^−1^·dm^−1^ in CHCl_3_ (C = 1).

^1^H NMR (400 MHz, CDCl_3_, δ, ppm): 5.66–5.12 (m, 2H), 5.01–4.71 (m, 2H), 3.31–0.45 (m, 38H).
^13^C NMR (100 MHz, CDCl_3_, δ, ppm): 173.15–171.92, 133.42–131.48, 80.68–79.12, 53.62–13.52.
Polymer **MP4**

The polymer yield was 95%. M_w_ = 5.8 × 10^5^, M_w_/M_n_ = 2.9. [α]_D_^22^ = −11 deg·mL·g^−1^·dm^−1^ in CHCl_3_ (C = 1).

^1^H NMR (400 MHz, CDCl_3_, δ, ppm): 5.50–5.11 (m, 2H, HRC=CRH), 4.12–0.77 (m, 40H).
^13^C NMR (100 MHz, CDCl_3_, δ, ppm): 172.94–172.52, 133.20–132.26, 69.29–69.27, 53.58–52.59, 45.25–38.60, 33.20–33.00, 28.12–27.95, 25.98–25.96, 23.42–23.41, 19.05–18.95
Polymer **MP6**

The polymer yield was 92%. M_w_ = 2.69 × 10^5^, M_w_/M_n_ = 2.2. [α]_D_^22^ = +4 deg·mL·g^−1^·dm^−1^ in CHCl_3_ (C = 1).

^1^H NMR (400 MHz, CDCl_3_, δ, ppm): 5.54–5.13 (m, 2H), 4.81–4.64 (m, 2H), 3.53–0.76 (m, 38H).
^13^C NMR (100 MHz, CDCl_3_, δ, ppm): 172.93–171.85, 134.12–131.61, 76.16–75.43, 54.30–52.16, 46.07–44.27, 41.03–39.23, 33.73–32.86, 32.16–31.58, 27.12–26.25, 25.50–24.51, 22.95–22.43, 14.55–13.79, 9.97–9.39.

### 2.8. Synthesis of Polymer ***MP7***

The monomer **M1** (0.30 g, 0.66 mmol) was dissolved in 0.80 mL of absolute toluene. RuCl_3_·3H_2_O (3.5 mg, 0.011 mmol) was dissolved in 1.0 mL of EtOH (abs) and the solution was stirred for 15 min. After that, the solution of **M1** in toluene was added to the solution of RuCl_3_ in ethanol. The resulting mixture was heated for 48 h at 75 °C. Then, the reaction mixture was precipitated by methanol containing the inhibitor (2,2′-methylenebis(6-tert-butyl-4-methylphenol)). The polymer was separated, washed with several portions of methanol, and dried in vacuum. The polymer was twice reprecipitated by methanol from the chloroform solution and dried in vacuum to a constant weight. The polymer yield was 18% (52.5 mg). M_w_ = 2.9 × 10^5^, M_w_/M_n_ = 2.6. [α]_D_^22^ = −46 deg·mL·g^−1^·dm^−1^ in CHCl_3_ (C = 1). T_g_ = 81 °C.

^1^H NMR (400 MHz, CDCl_3_, δ, ppm): 5.54–5.37 (m, 2H), 4.75–458 (m, 2H), 3.10–0.55 (m, 42H).

## 3. Results and Discussion

### 3.1. Synthesis of Monomers

**Scheme 1:**
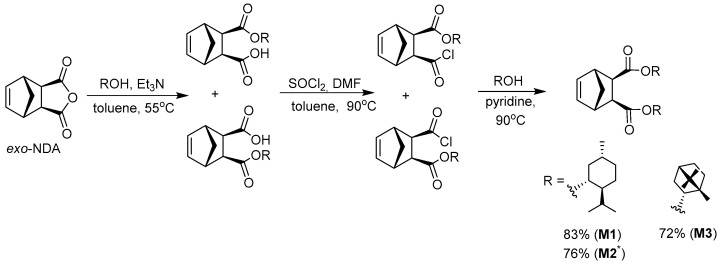
The synthesis of monomers **M1–M3** (*M2 is *endo*-isomer of **M1**).

**Scheme 2:**
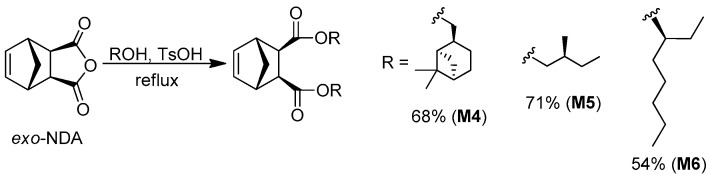
The synthesis of monomers **M4–M6**.

Herein we designed and synthesized two sets of new chiral di-substituted norbornene-type monomers (Scheme 1 and Scheme 2). The chiral groups in these monomers are attached to norbornene moiety via ester linkages. As the source of norbornene moiety, *cis*-5-norbornene-*exo*-2,3-dicarboxylic anhydride (*exo*-NDA) was chosen due to its preparative availability and high reactivity. As starting materials for introducing chiral fragments, renewable optically active alcohols ((-)-menthol, (-)-borneol, and pinanol), as well as commercial chiral primary and secondary alcohols, were used.

The required monomers were readily prepared in moderate or good yields according to one- and three-step procedures (Table 1). Primary and less sterically hindered alcohols reacted directly with *exo*-NDA with the formation of the corresponding diesters (Scheme 2). This scheme was found to be inefficient for the preparation of diesters of *cis*-5-norbornene-*exo*-2,3-dicarboxylic acid based on (-)-menthol and (-)-borneol. Therefore, an alternative way for the synthesis of the corresponding diesters from (-)-menthol and (-)-borneol was realized (Scheme 1). By the usage of this scheme, a diester **M2**, which is an isomer of **M1**, was also obtained from *endo*-NDA and (-)-menthol to evaluate the influence of *exo-/endo*-isomerism on the properties of resulting polymers. It is worth noting that intermediates formed in the first step of the three-step procedure can be isolated as mixtures of the diastereomers (Scheme 1).

All the synthesized monomers were isolated as individual compounds. Their structure and purity were analyzed by ^1^H, ^13^C NMR spectroscopy (Figures S1–S11), and HRMS (Figures S13–S18). In the case of solid monomers **M1** and **M2**, X-ray analysis was additionally performed (Figure 2, Table S1), which confirmed the expected structures of the studied monomers. The specific optical rotations for the monomers were nonzero (Table 1). The optical activity of these compounds was also confirmed by circular dichroism (CD) spectroscopy, showing a Cotton effect (Figures S31–S35).

**Scheme 3:**
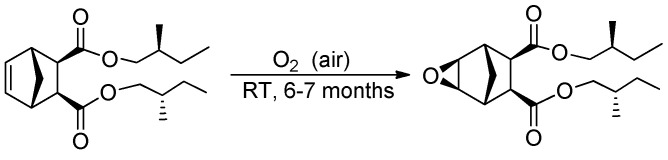
The oxidation of **M5** during storage in air.

**Figure 3:**
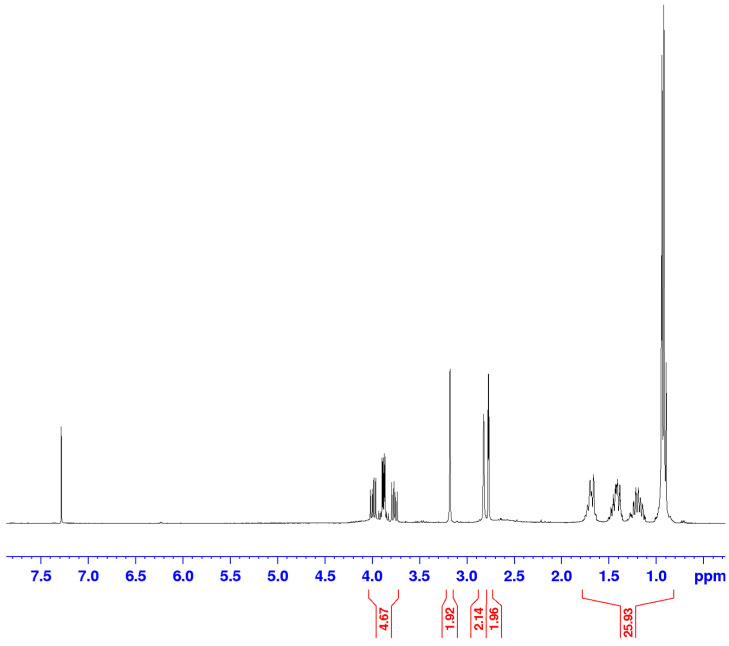
^1^H NMR spectrum of the epoxide based on **M5** (CDCl_3_).

Interestingly, the synthesized diesters showed a tendency to be slowly oxidized with atmospheric oxygen. We found that **M5** was oxidized with atmospheric oxygen for several months, affording the corresponding epoxide in the quantitative yield (Scheme 3, Figure 3 and Figure S12). This is different from the related N-substituted imides of *cis*-5-norbornene-*exo*-2,3-dicarboxylic acid which are stable in the air [41]. Therefore, it is desirable to store such diesters in an inert atmosphere.

### 3.2. Ring-Opening Metathesis Polymerization

**Scheme 4:**
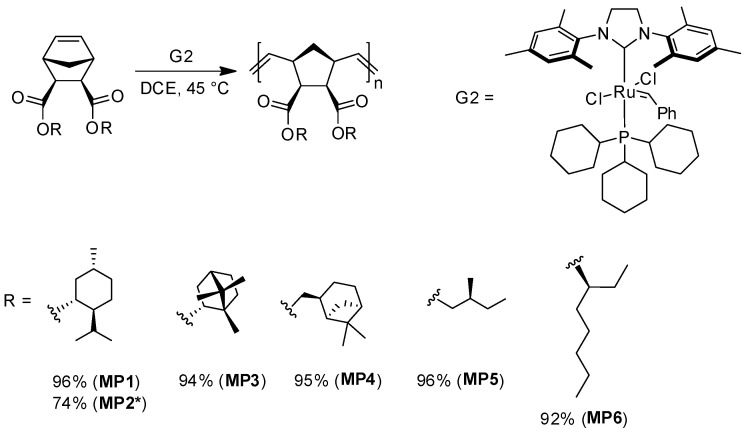
Ring-opening metathesis polymerization of the prepared monomers (*MP2 was obtained from **M2** (*endo*-isomer of **M1)**).

Ring-opening metathesis polymerization of the synthesized monomers was studied in the presence of the second-generation Grubbs catalyst (Scheme 4). All the monomers exhibited high reactivity. ROMP polymerization of diesters of *cis*-5-norbornene-*exo*-2,3-dicarboxylic acid proceeded for a couple of hours at catalyst loadings of 0.1–0.2 mol.% with the formation of soluble high-molecular weight products in good and high yields (Table 2). For the synthesized polymers, relatively narrow values of molecular weight distributions were found.

**Figure 4:**
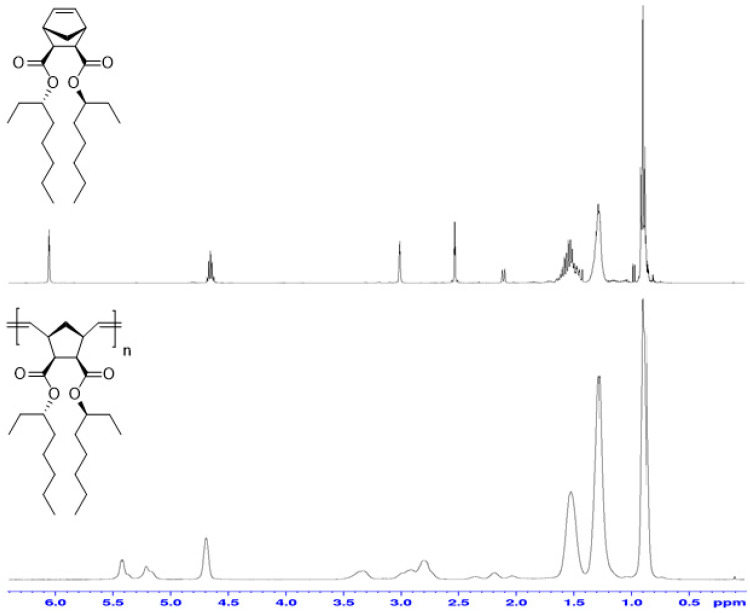
^1^H NMR spectra of **M6** and the corresponding ROMP polymer from **M6** (CDCl_3_).

**Figure 5:**
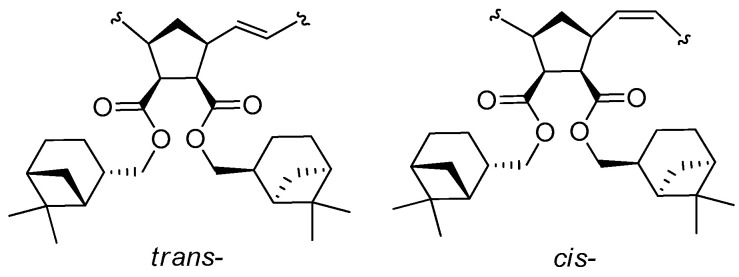
The structure of monomer units of **MP4** with *trans-* and *cis*-double bonds.

**Figure 6:**
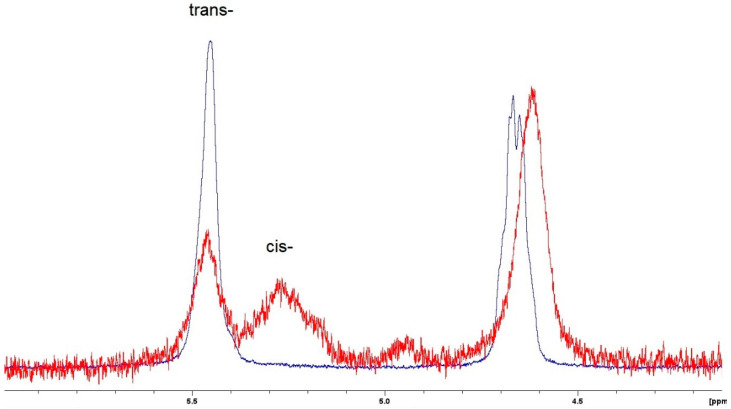
The parts of ^1^H NMR spectra of the isomeric ROMP polymers obtained from **M1**: **MP1** (red curve) and **MP7** (blue curve).

The reactivity of the *endo*-isomer was noticeably lower than that of the *exo*-isomer (**M2** vs. **M1**, Table 2). Nevertheless, we succeeded to obtain ROMP polymer from **M2** with high M_w_ (above 1 × 10^5^) in a good yield. The analysis of the prepared ROMP polymers with NMR-spectroscopy confirmed the expected structure (Figure 4 and Figures S19–S30). The *cis-/trans*-ratio of double bonds in monomer units of these polymers (Figure 5 and Figure 6) was analyzed with ^1^H NMR spectroscopy. The signals of protons at *cis*- and *trans*-double bonds for these polymers are at around 5.2 ppm and 5.35 ppm, correspondingly. This was confirmed by the synthesis of the isomeric ROMP polymer from **M1** (polymer **MP7**, Figure 6) in the presence of RuCl_3_·3H_2_O/ethanol catalytic system, which gives ROMP polymers with predominantly *trans*-double bonds (>90%). The studied ROMP polymers obtained over the second generation Grubbs catalyst contained a little more *cis*- than *trans-*double bonds (53–60%, e.g., the *cis-/trans-*double bond ratio was 54/46 for **MP1**).

These ROMP polymers are optically active as it was established by the measurements of the specific optical rotations (Table 2) and by CD spectroscopy (Figures S31–S35). The *cis/trans*-ratio of double bonds has an effect on optical activity. For instance, **MP1** and **MP7** are isomeric ROMP polymers differing in the ratio of *cis/trans*-double bonds (>98% *trans*-double bonds in **MP7** and 46% of *trans*-double bonds in **MP1**). The specific optical rotations are −59 deg·mL·g^−1^·dm^−1^ and −46 deg·mL·g^−1^·dm^−1^ for **MP1** and **MP7**, correspondingly. It should be noted that the *cis/trans*-ratio of double bonds can affect the secondary structure of the obtained polymers, forming, for example, a helix in the case of polymers containing predominantly one type of double bond. This study is in progress now.

**Figure 7:**
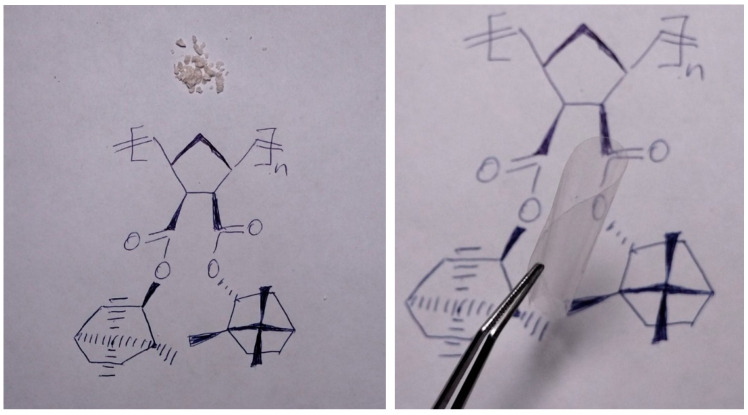
The photographs of the powder and thin films of **MP3**.

The resulting ROMP polymers showed good solubility in THF and chloroform while swelling in many other common organic solvents (Table 3). The good solubility and high molecular weights allowed preparing free-standing and robust thin films from these polymers (Figure 7).

The density of the obtained polymers exceeded 1.0 g/cm^3^ (Table 4), which is unusual because, for related halogen-free polymers, the density is typically less than 1 g/cm^3^. Despite the high density, the fraction free volume (FFV) for these polymers is in the range of 16–17%, which is higher than the values typical for polymers with low free volume [42]. It is possible that the obtained FFV values can be explained by the presence of bulky and rigid side-substituents in such polymers, which create free-volume elements near them.

The synthesized polymers showed glass transition temperatures (T_g_) in the range of −30 to +139 °C (Table 2), depending on the substituents’ nature. At the same time, there were no melting peaks in the DSC curves (Figure S36), which indicates the amorphous nature of the prepared polymers. The ROMP polymers with long flexible alkyl groups had the lowest values of T_g_, while the highest T_g_ was observed for the ROMP polymer with bulky and rigid borneol moieties in side chains. Interestingly, isomeric polymers based on *exo*-NDA and *endo*-NDA diesters (**MP1** vs. **MP2**) showed very different T_g_ values, and **MP2** derived from *endo*-NDA had a higher T_g_ value, evidencing that **MP2** possesses more rigid polymeric backbones.

**Figure 8:**
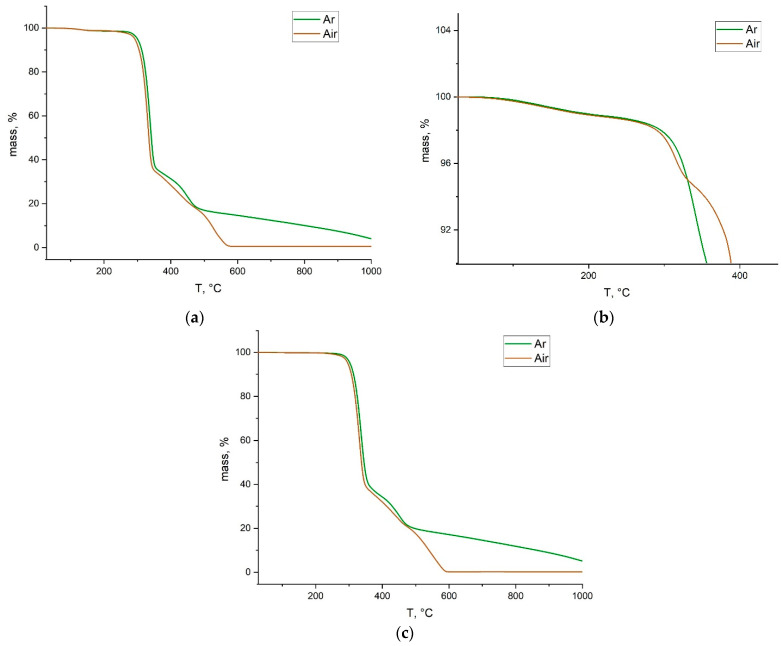
TGA curves for **MP1** (**a**), the enlarged TGA curves for **MP1** (**b**), and TGA curves for **MP6** (**c**).

TGA analysis of the synthesized ROMP polymers showed their high thermal stability (Figure 8, Figures S37–S42). The decomposition temperatures (5 wt.% loss) in argon and in air were close and exceeded 300 °C. For some of the resulting polymers, a small weight loss (1–2%) was observed around 180–200 °C, probably due to traces of sorbed water (Figure 8b). Char yields at 1000 °C were close to zero, which indicates the almost complete decomposition of the polymers at high temperatures.

**Figure 9:**
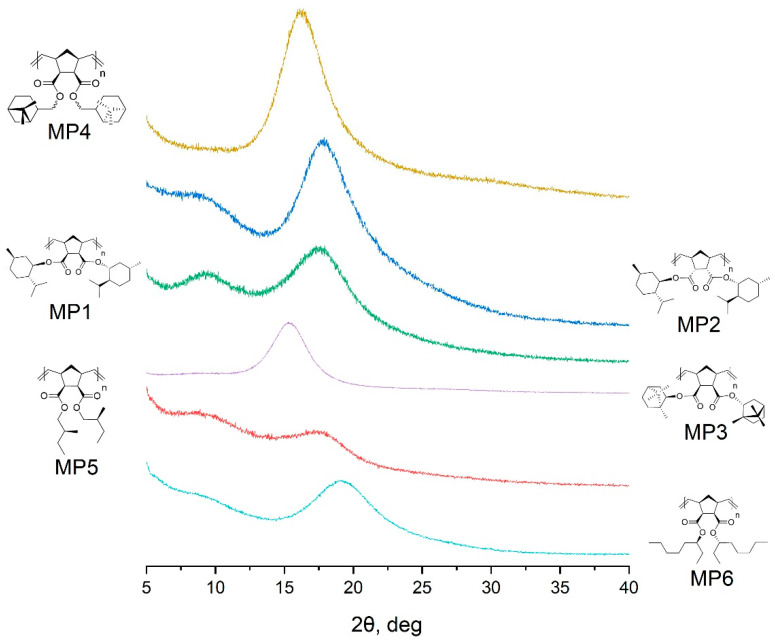
Wide-angle X-ray diffraction (WAXD) patterns for the prepared ROMP polymers.

The state of polymers is another important characteristic that determines the performance of a polymer in many applications. According to the WAXD study, all the synthesized polymers are amorphous (Figure 9). WAXD patterns for **MP1**, **MP2**, **MP5** and **MP6** are represented by two broad signals. At the same time, for **MP3** and **MP4** the second peak was not observed and WAXD patterns are represented by one intensive broad peak at larger 2θ angles. The WAXD patterns of **MP1** were the same before and after heating the polymer sample, and the intensity of the peak in WAXD patterns only slightly changed after heating (Figure S43), confirming the amorphous nature of the polymer. It is worth noting that usually there is a single broad peak for conventional amorphous glassy polymers in their WAXD patterns. Two signals in WAXD patterns of **MP1**, **MP2**, **MP5** and **MP6** are not usual for amorphous glassy polymers. It was shown that the signals at higher 2θ angles are related to intrasegmental interactions and the signals at lower 2θ angles to intersegmental interactions [44,45]. Therefore, it seems that both intra- and inter-segmental interactions are significant in the case of **MP1**, **MP2**, **MP5** and **MP6**. Within this group of polymers, d_1_- and d_2_-distances are practically the same (Table 5). In turn, **MP3** and **MP4** had d_1_-distances close to d_2_-distances for **MP1**, **MP2**, **MP5** and **MP6** polymers. It should be noted, although there is no clear trend between WAXD data and real porosity, the polymers with larger d-spacings often have a more porous structure [46]. Thus, a more porous structure in the case of **MP1**, **MP2**, **MP5** and **MP6** can be expected.

The amorphous nature of the synthesized polymers, good film-forming properties, and high thermal stability, combined with the facile method of the synthesis, make these polymers attractive for the detailed study of their properties in the future as promising materials for dense enantioselective membranes and chiral stationary phases for chromatography.

## 4. Conclusions

A set of new optically active polymers was developed based on chiral norbornene-type monomers. The desired monomers were readily prepared in good yields by the esterification of *cis*-5-norbornene-2,3-dicarboxylic anhydride with alcohols from the renewable sources ((-)-menthol, (-)-borneol, and pinanol) and with commercially available alcohols (S-(-)-2-methylbutanol-1, S-(+)-3-octanol). ROMP polymerization of these monomers over the second-generation Grubbs catalyst afforded the metathesis polymers in good yields with high-molecular weights. The synthesized ROMP polymers showed high thermal stability. They had good film-forming properties, giving free-standing thin films from solutions suitable for studying membrane properties. The properties of the polymers strongly depended both on the nature of side groups and the type of their bonding with monomer units (*exo-/endo*-isomerism). Glass transition temperatures for the prepared polymers can be varied in a wide range (from −30 °C to +139 °C) and, therefore, the state of the polymers can be changed from rubbery to glassy. Thus, it is possible to fine-tune the properties of these polymers to achieve the desired characteristics by changing the nature of the side chains. The prepared polymers represent a new attractive platform of chiral polymeric materials for enantioselective membrane separation and chiral stationary phases for chromatography.

## Figures and Tables

**Figure 2:**
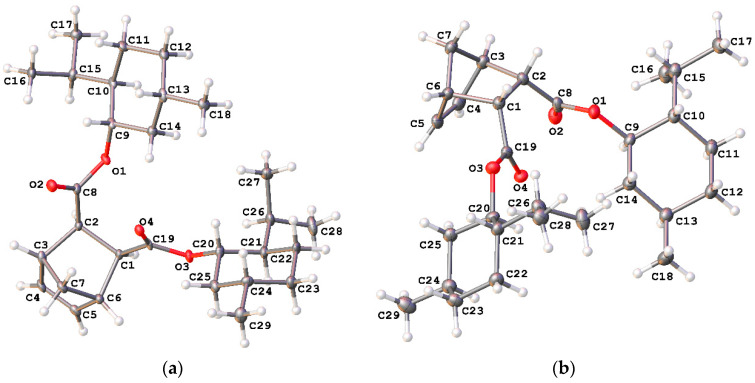
The structure of **M1** (**a**) and **M2** (**b**) according to X-ray analysis.

**Table 1:** The data on the synthesis and some properties of the monomers.

Monomer	Reaction Conditions ^a^	Yield, %	[α]_D_^b^, deg·mL·g^−1^·dm^−1^	T_m_, °C
**M1**	*i*	83	−75 (T = 25 °C, CHCl_3_)	86–89
**M2**	*i*	76	−125 (T = 20 °C, CHCl_3_)	97–100
**M3**	*i*	72	−42 (T = 25 °C, CHCl_3_)	96–98
**M4**	*ii*	68	−11 (T = 20 °C, CHCl_3_)	Colorless oil
**M5**	*ii*	71	+7 (T = 20 °C, THF)	Colorless oil
**M6**	*ii*	54	+4 (T = 20 °C, CHCl_3_)	Colorless oil

^a^: *i*—three-step procedure, (1) alcohol (4.5 eq.), Et_3_N, toluene, 55 °C. (2) SOCl_2,_ DMF (few drops)_,_ toluene, 90 °C. (3) alcohol (0.9 eq.), pyridine, 90 °C; *ii*—one-step procedure, alcohol (1.05 eq.), toluene, TsOH, reflux. ^b^: the specific optical rotation was measured at C = 1.

**Table 2:** ROMP polymerization of diesters of *cis*-5-norbornene-2,3-dicarboxylic acid over the 2nd generation Grubbs catalyst ^a^.

Polymer	Monomer/[Ru] Molar Ratio	C, M ^b^	Yield, %	M_w_ × 10^−3^	M_w_/M_n_^c^	T_g_, °C ^d^	[α]_D_^e^, deg·mL·g^−1^·dm^−1^
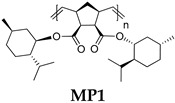	1000/1	0.5	96	488	2.1	80	−59
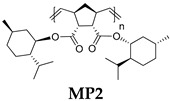	750/1	0.5	74	190	1.6	136	−64
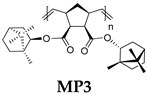	500/1	0.5	94	469	2.1	139	−34
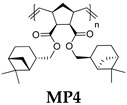	1000/1	0.5	95	582	2.9	68	−11
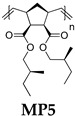	500/1	0.5	96	457	2.2	−9	+10 ^f^
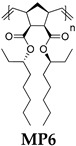	500/1	0.5	92	269	2.2	−30	+4

^a^—reaction temperature—+45 °C, solvent—1,2-dichloroethane, [Ru]—the 2nd generation Grubbs catalyst; ^b^—the initial concentration of a monomer; ^c^—the molecular weights of polymers were estimated by means of gel permeation chromatography in THF; ^d^—glass transition temperature according to DSC; ^e^—the specific optical rotation was measured at 22 °C in chloroform solution at C = 1; ^f^—the specific optical rotation was measured in THF solution.

**Table 3:** Solubility of the ROMP polymers synthesized from diesters of *cis*-5-norbornene-2,3-dicarboxylic acid ^a^.

Polymer	CH_3_CN	Acetone	DMA	DMSO	*n*-Hexane	PhCH_3_	THF	CHCl_3_
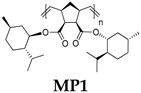	±	±	±	–	+	±	+	+
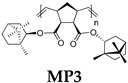	–	±	±	–	±	+	+	+
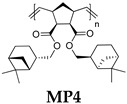	±	±	±	±	±	+	+	+
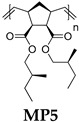	±	±	±	±	±	+	+	+
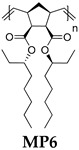	–	+	±	±	±	+	+	+

*^a^—*“+”—soluble, “–”—insoluble, and “±”—swelling.

**Table 4:** Density and fractional free-volume (FFV) for ROMP polymers synthesized from diesters of *cis*-5-norbornene-2,3-dicarboxylic acid ^a^.

Polymer	Density, g/cm^3^	FFV, %
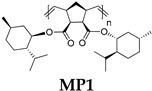	1.033	16
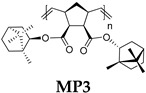	1.064	17
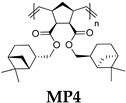	1.110	13

^a^—the density of polymer films was determined by the hydrostatic weighing; fractional free-volume was calculated by Bondi’s method [43] from the density of polymer films.

**Table 5:** The intersegmental and intrasegmental distances (*d*, Å) evaluated with Bragg-formula for the prepared ROMP polymers.

Polymer	2*θ*_1_,°	*d*_1_, Å	*2θ*_2_,°	*d*_2_, Å
**MP1**	9.2	9.6	17.6	5.0
**MP2**	9.1	9.7	17.8	5.0
**MP3**	15.2	5.8	-	-
**MP4**	15.9	5.6	-	-
**MP5**	9.3	9.5	17.6	5.0
**MP6**	9.1	9.7	19.0	4.7

## Data Availability

NMR, CD spectra, and TGA curves of the monomers and the polymers are available free of charge as a file of Supplementary Materials.

## Supplementary Materials

The following supporting information can be downloaded at: https://www.mdpi.com/article/10.3390/polym14245453/s1, Figures S1–S12: NMR spectra of monomers; Figures S13–S18: HRMS spectra of monomers; Figures S19–S30: NMR spectra of polymers; Figures S31–S35: CD spectra of monomers and polymers; Figures S36–S42: DSC and TGA curves for polymers; Figure S43: WAXD patterns of MP1; Table S1: Crystal data and structure refinement details for M1 and M2.
