# Supplementary material for: Chiral Polymers from Norbornenes Based on Renewable Chemical Feedstocks
Source: Polymers (Basel). 2022 Dec 13;14(24):5453. doi: 10.3390/polym14245453 (PMC9786787; doi:10.3390/polym14245453)

checkCIF/PLATON report

Structure factors have been supplied for datablock(s) NZR3711

THIS REPORT IS FOR GUIDANCE ONLY. IF USED AS PART OF A REVIEW PROCEDURE FOR PUBLICATION, IT SHOULD NOT REPLACE THE EXPERTISE OF AN EXPERIENCED CRYSTALLOGRAPHIC REFEREE.

No syntax errors found. CIF dictionary Interpreting this report

Datablock: NZR3711

```
Bond precision:   C-C = 0.0065 A          Wavelength=1.54178

Cell:            a=23.4647(18)     b=5.7765(4)         c=21.9950(17)
                 alpha=90          beta=115.155(4)     gamma=90
Temperature:     120 K

                 Calculated                   Reported
Volume           2698.6(4)                    2698.5(4)
Space group      C 2                          C 1 2 1
Hall group       C 2y                         C 2y
Moiety formula   C29 H46 O4                   C29 H46 O4
Sum formula      C29 H46 O4                   C29 H46 O4
Mr               458.66                       458.66
Dx,g cm-3        1.129                        1.129
Z                4                            4
Mu (mm-1)        0.571                        0.571
F000             1008.0                       1008.0
F000'            1010.78
h,k,lmax         27,6,25                      27,6,25
Nref             4599[ 2552]                  4051
Tmin,Tmax        0.966,0.977                  0.512,0.753
Tmin'            0.944

Correction method= # Reported T Limits: Tmin=0.512 Tmax=0.753
AbsCorr = MULTI-SCAN

Data completeness= 1.59/0.88        Theta(max)= 64.951

R(reflections)= 0.0499( 3131)                          wR2(reflections)=
                                                       0.1461( 4051)
S = 1.069                    Npar= 306
```

The following ALERTS were generated. Each ALERT has the format
test-name_ALERT_alert-type_alert-level.
Click on the hyperlinks for more details of the test.

Alert level C

```
THETM01_ALERT_3_C  The value of sine(theta_max)/wavelength is less than 0.590
            Calculated sin(theta_max)/wavelength =    0.5876
PLAT018_ALERT_1_C _diffrn_measured_fraction_theta_max .NE. *_full           ! Check
PLAT230_ALERT_2_C Hirshfeld Test Diff for    O2        --C8        .        5.3 s.u.
PLAT340_ALERT_3_C Low Bond Precision on  C-C Bonds ...............    0.00647 Ang.
PLAT911_ALERT_3_C Missing FCF Refl Between Thmin & STh/L=    0.588         90 Report
PLAT915_ALERT_3_C No Flack x Check Done: Low Friedel Pair Coverage         78 %
```

Alert level G

```
PLAT032_ALERT_4_G Std. Uncertainty on Flack Parameter Value High .      0.400 Report
PLAT791_ALERT_4_G Model has Chirality at C1          (Sohnke SpGr)          R Verify
PLAT791_ALERT_4_G Model has Chirality at C2          (Sohnke SpGr)          S Verify
PLAT791_ALERT_4_G Model has Chirality at C3          (Sohnke SpGr)          R Verify
PLAT791_ALERT_4_G Model has Chirality at C6          (Sohnke SpGr)          S Verify
PLAT791_ALERT_4_G Model has Chirality at C9          (Sohnke SpGr)          R Verify
PLAT791_ALERT_4_G Model has Chirality at C10         (Sohnke SpGr)          S Verify
PLAT791_ALERT_4_G Model has Chirality at C13         (Sohnke SpGr)          R Verify
PLAT791_ALERT_4_G Model has Chirality at C20         (Sohnke SpGr)          R Verify
PLAT791_ALERT_4_G Model has Chirality at C21         (Sohnke SpGr)          S Verify
PLAT791_ALERT_4_G Model has Chirality at C24         (Sohnke SpGr)          R Verify
PLAT850_ALERT_4_G Check Flack Parameter Exact Value 0.00 with s.u.       0.40 Check
PLAT909_ALERT_3_G Percentage of I>2sig(I) Data at Theta(Max) Still        39% Note
PLAT933_ALERT_2_G Number of HKL-OMIT Records in Embedded .res File          1 Note
PLAT967_ALERT_5_G Note: Two-Theta Cutoff Value in Embedded .res ..      130.0 Degree
PLAT978_ALERT_2_G Number C-C Bonds with Positive Residual Density.          0 Info
```

0 **ALERT level A** = Most likely a serious problem - resolve or explain
0 **ALERT level B** = A potentially serious problem, consider carefully
6 **ALERT level C** = Check. Ensure it is not caused by an omission or oversight
16 **ALERT level G** = General information/check it is not something unexpected

1 ALERT type 1 CIF construction/syntax error, inconsistent or missing data
3 ALERT type 2 Indicator that the structure model may be wrong or deficient
5 ALERT type 3 Indicator that the structure quality may be low
12 ALERT type 4 Improvement, methodology, query or suggestion
1 ALERT type 5 Informative message, check

It is advisable to attempt to resolve as many as possible of the alerts in all categories. Often the minor alerts point to easily fixed oversights, errors and omissions in your CIF or refinement strategy, so attention to these fine details can be worthwhile. In order to resolve some of the more serious problems it may be necessary to carry out additional measurements or structure refinements. However, the purpose of your study may justify the reported deviations and the more serious of these should normally be commented upon in the discussion or experimental section of a paper or in the "special_details" fields of the CIF. checkCIF was carefully designed to identify outliers and unusual parameters, but every test has its limitations and alerts that are not important in a particular case may appear. Conversely, the absence of alerts does not guarantee there are no aspects of the results needing attention. It is up to the individual to critically assess their own results and, if necessary, seek expert advice.

Publication of your CIF in IUCr journals

A basic structural check has been run on your CIF. These basic checks will be run on all CIFs submitted for publication in IUCr journals (*Acta Crystallographica*, *Journal of Applied Crystallography*, *Journal of Synchrotron Radiation*); however, if you intend to submit to *Acta Crystallographica Section C* or *E* or *IUCrData*, you should make sure that full publication checks are run on the final version of your CIF prior to submission.

Publication of your CIF in other journals

Please refer to the *Notes for Authors* of the relevant journal for any special instructions relating to CIF submission.

PLATON version of 12/09/2022; check.def file version of 09/08/2022

Datablock NZR3711 - ellipsoid plot

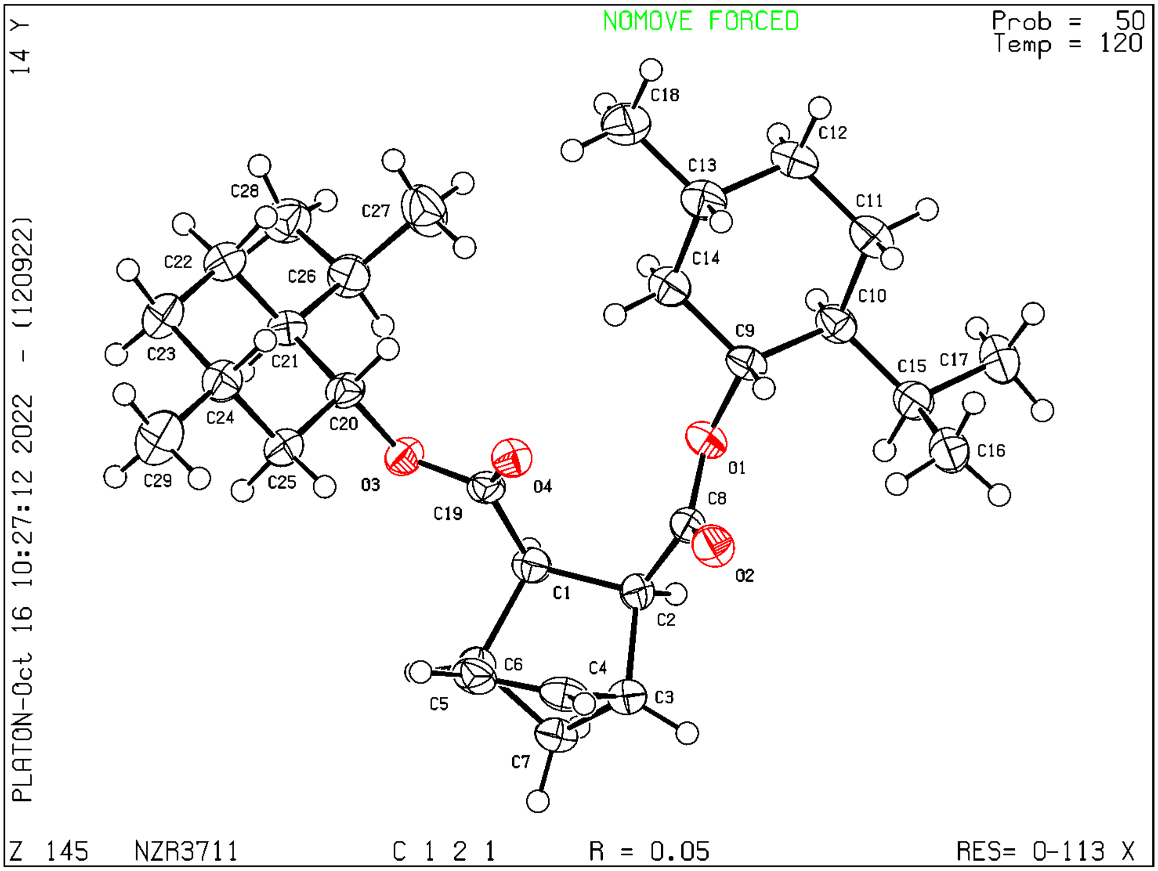

Supplement: Supplementary file 1 [file polymers-14-05453-s001.zip › checkcif_NZR3711.pdf]
