# Supplementary material for: Chiral Polymers from Norbornenes Based on Renewable Chemical Feedstocks
Source: Polymers (Basel). 2022 Dec 13;14(24):5453. doi: 10.3390/polym14245453 (PMC9786787; doi:10.3390/polym14245453)
Supplement: Supplementary file 1 [file polymers-14-05453-s001.zip › polymers-2047226-supplementary.pdf]

Supplementary Material

Chiral polymers from norbornenes based on renewable chemical feedstocks

Ivan V. Nazarov [1], Danil P. Zarezin [1], Ivan A. Solomatov [1], Anastasya A. Danshina [2,3], Yulia V. Nelyubina [2],
Igor R. Ilyasov [4] and Maxim V. Bermeshev [1,*]

[1] A.V. Topchiev Institute of Petrochemical Synthesis, RAS, 29 Leninskiy Pr., 119991 Moscow, Russia
[2] A.N. Nesmeyanov Institute of Organoelement Compounds, Russian Academy of Sciences, Vavilov Street 28, 119991 Moscow, Russia
[3] Moscow Institute of Physics and Technology, National Research University, Institutskiy Per., 9, 141700 Dolgoprudny, Russia
[4] Nelubin Institute of Pharmacy, Sechenov First Moscow State Medical University, Trubetskaya Str. 8/2, 119991 Moscow, Russia
* Correspondence: bmv@ips.ac.ru; Tel.: +7-495-647-59-27 (ext.379)

Table of contents

NMR spectra of the monomers....................2
ESI-HRMS spectra of the monomers....................9
X-ray diffraction data for M1 and M2....................12
NMR spectra of the ROMP polymers....................13
CD spectra of the monomers and the ROMP polymers....................20
DSC curves of the polymers....................23
TGA curves of the ROMP polymers....................24
WAXD study of MP1....................27

NMR spectra of the monomers

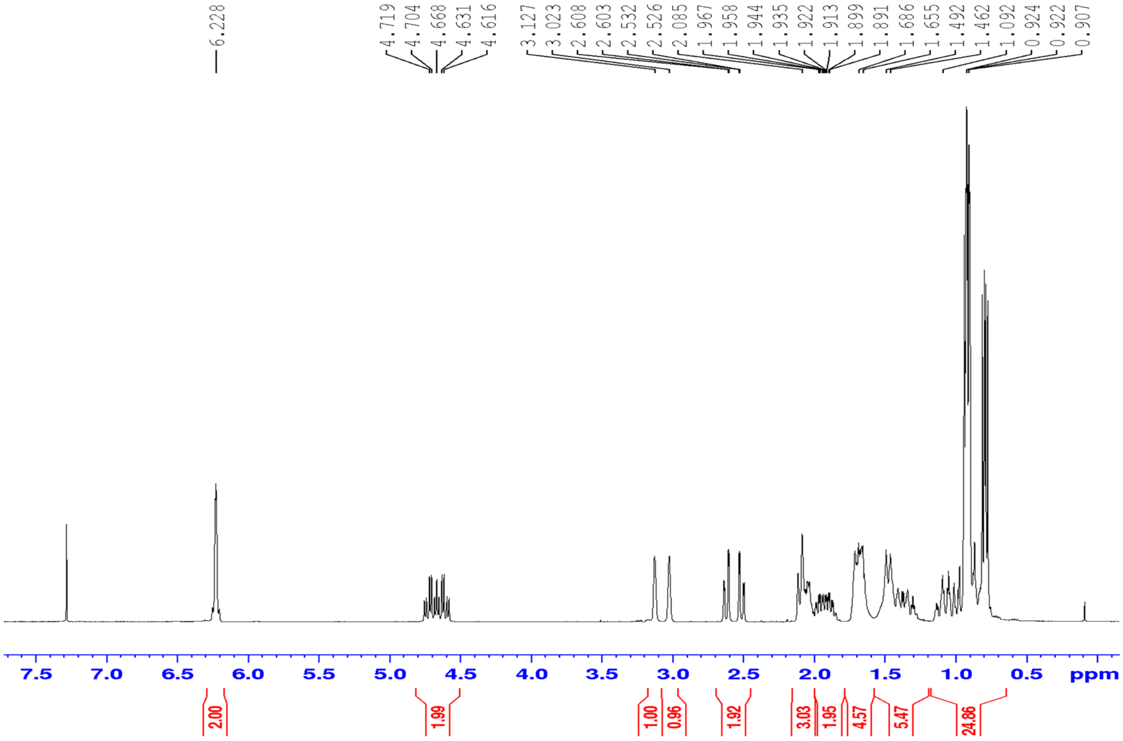

Figure S1. ^{1}H NMR spectrum of **M1** ($CDCl_3$).

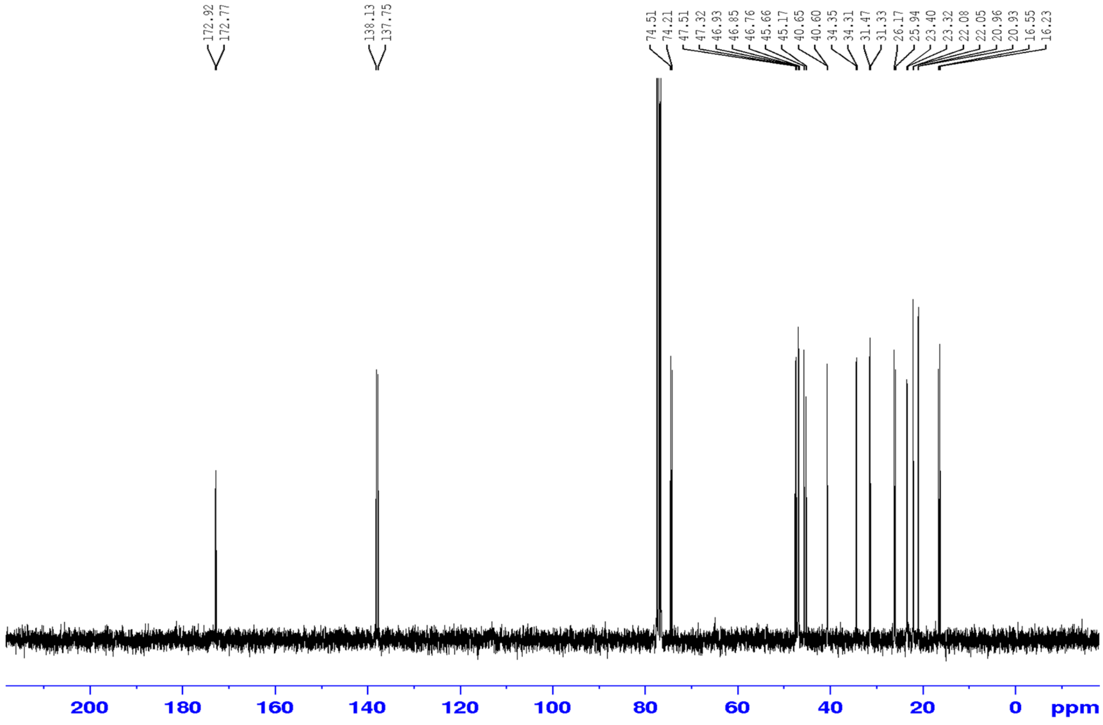

Figure S2. ^{13}C NMR spectrum of **M1** ($CDCl_3$).

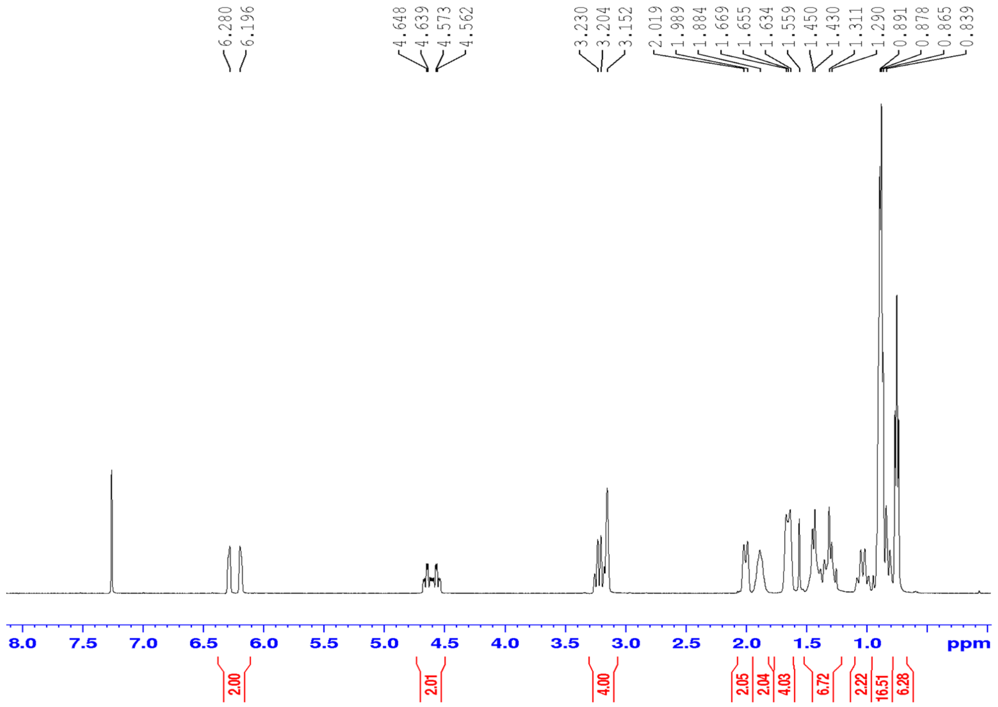

Figure S3. ^{13}C NMR spectrum of **M2** ($CDCl_3$).

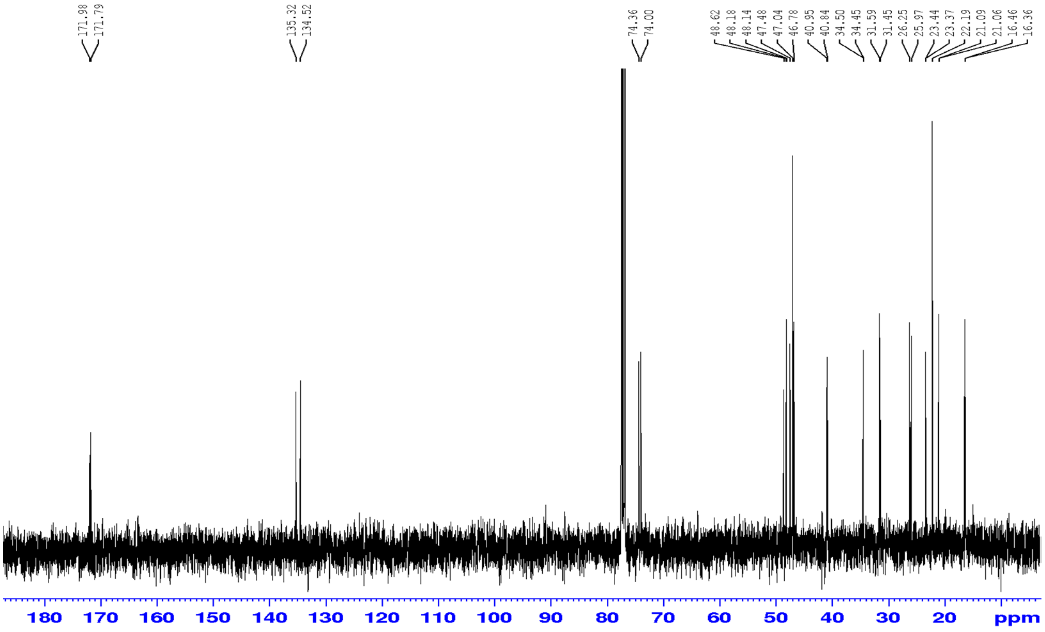

Figure S4. ^{13}C NMR spectrum of **M2** ($CDCl_3$).

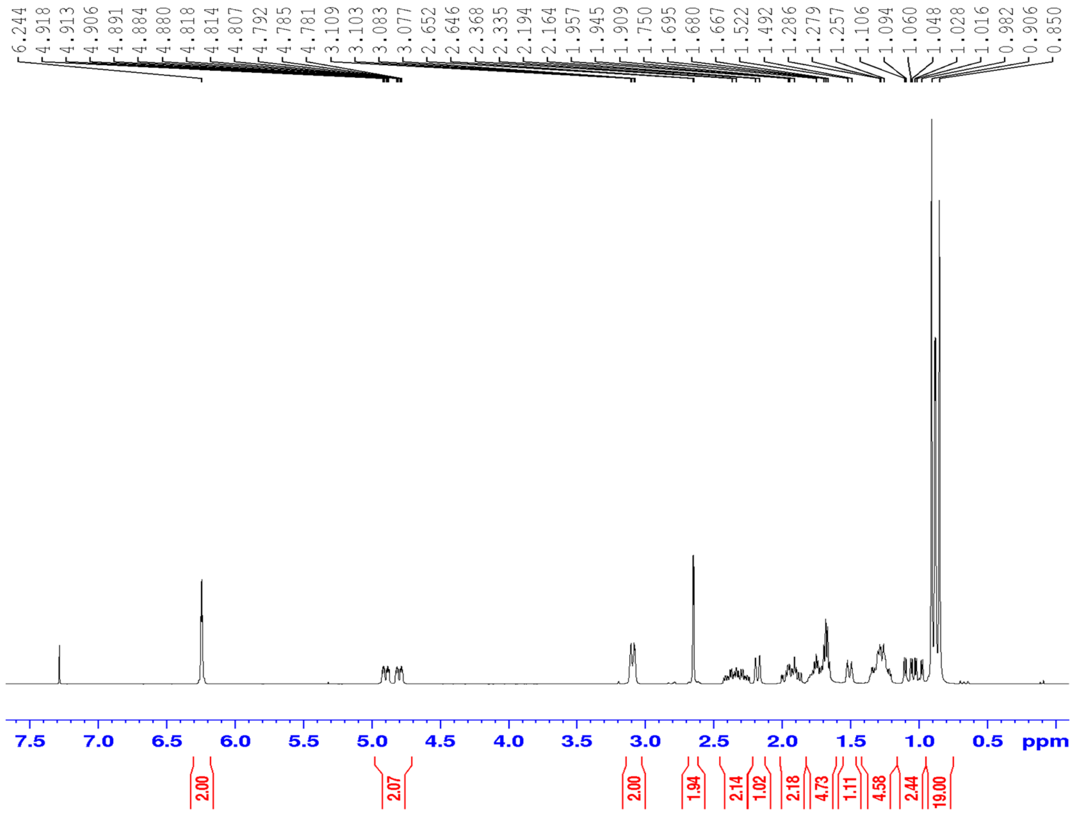

Figure S5. ^{1}H NMR spectrum of **M3** ($CDCl_3$).

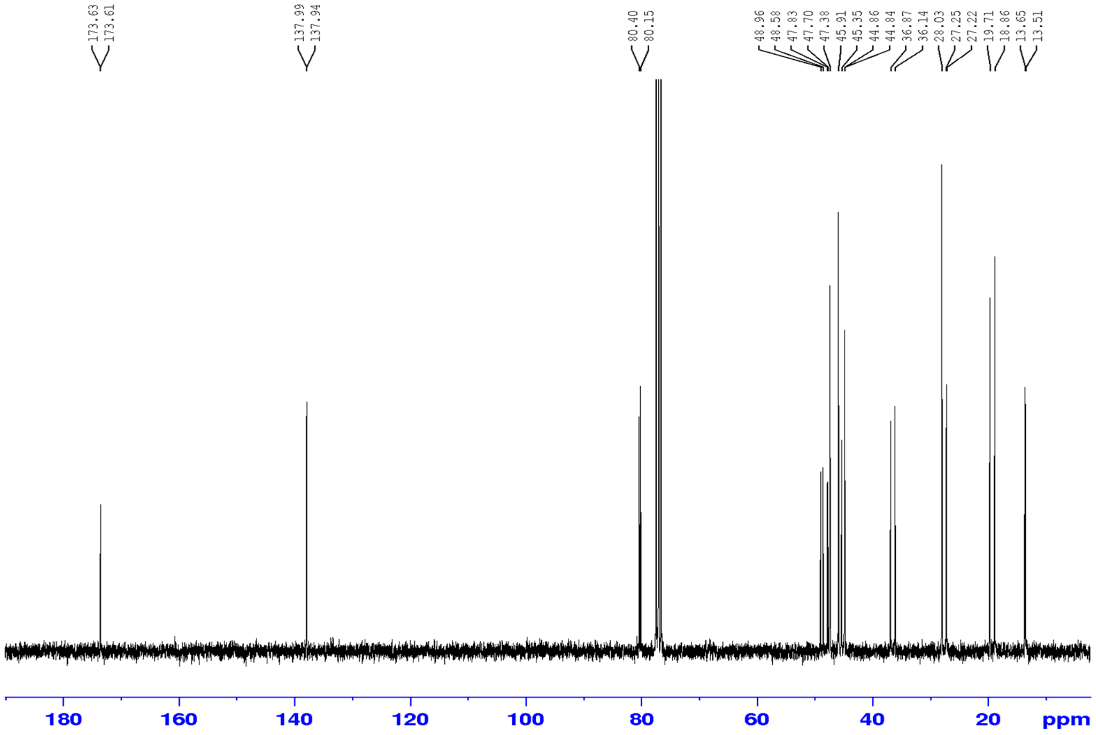

Figure S6. ^{13}C NMR spectrum of **M3** ($CDCl_3$).

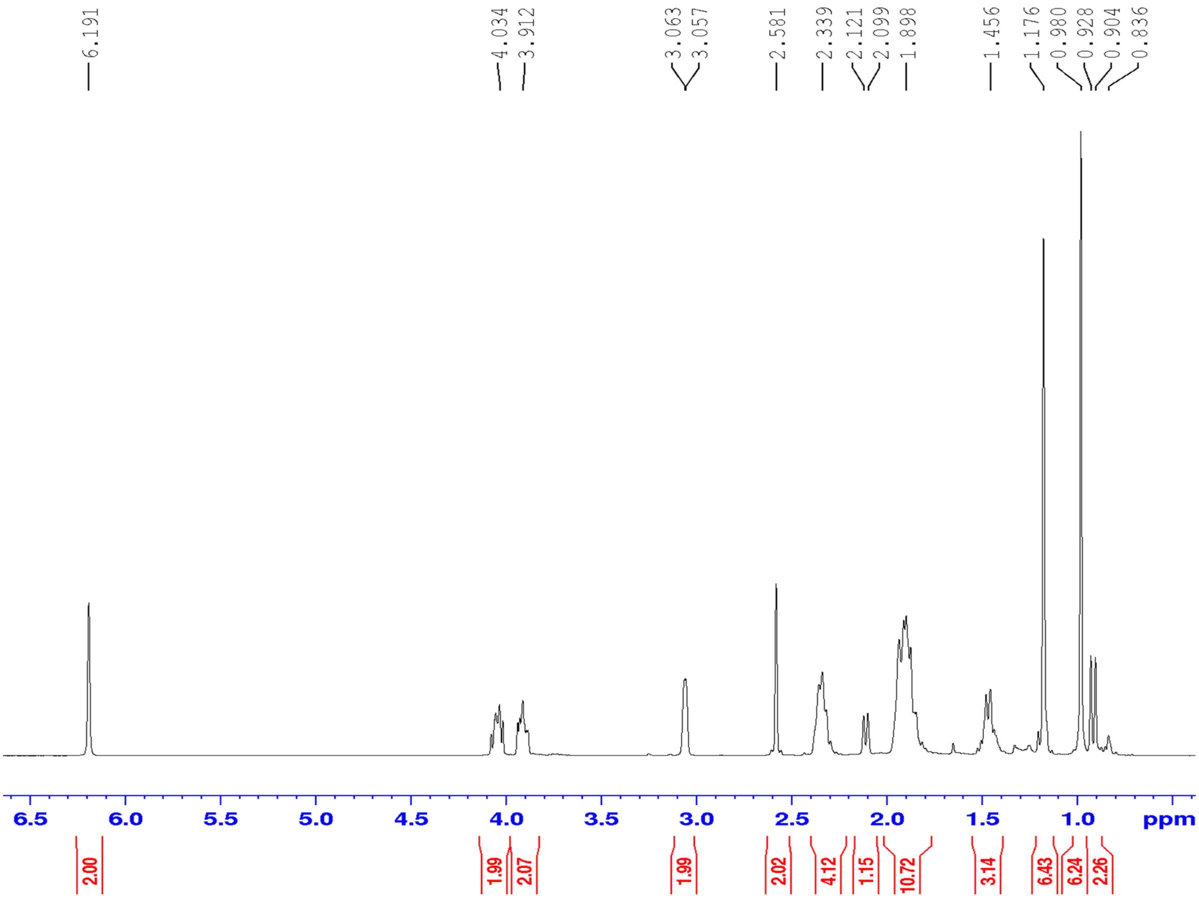

Figure S7. ^{1}H NMR spectrum of **M4** ($CDCl_3$).

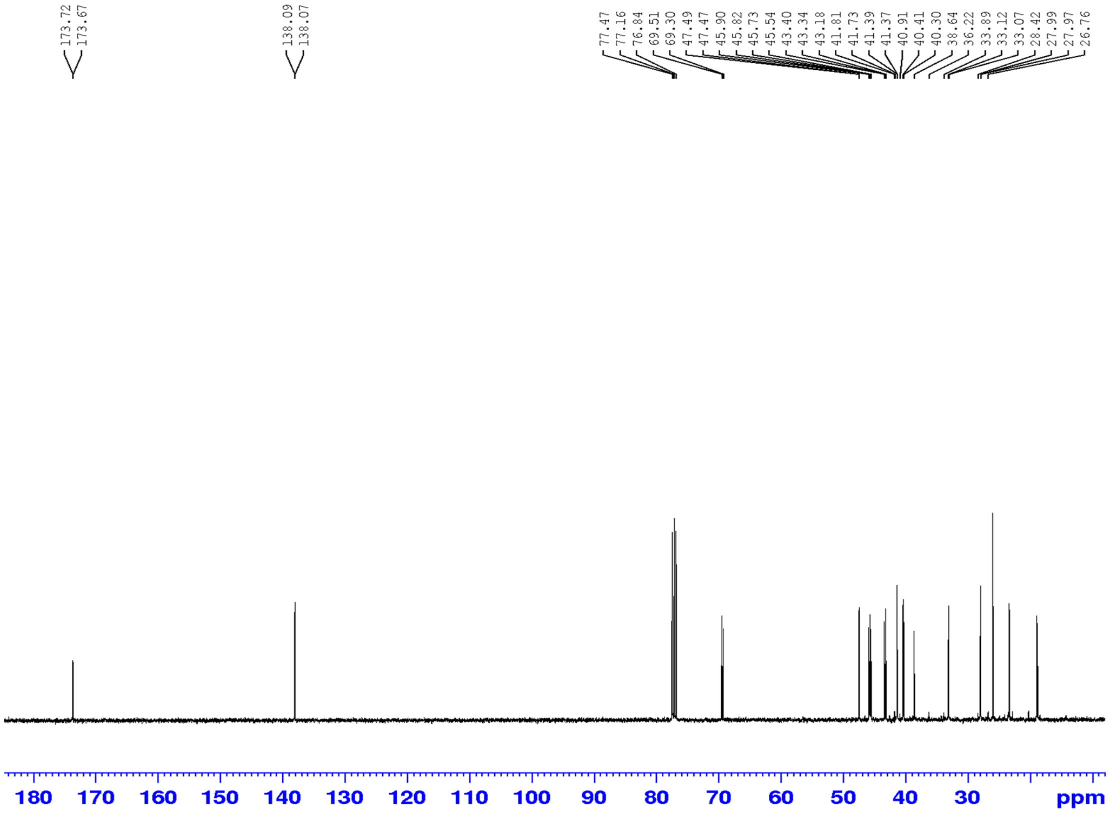

Figure S8. ^{13}C NMR spectrum of **M4** ($CDCl_3$).

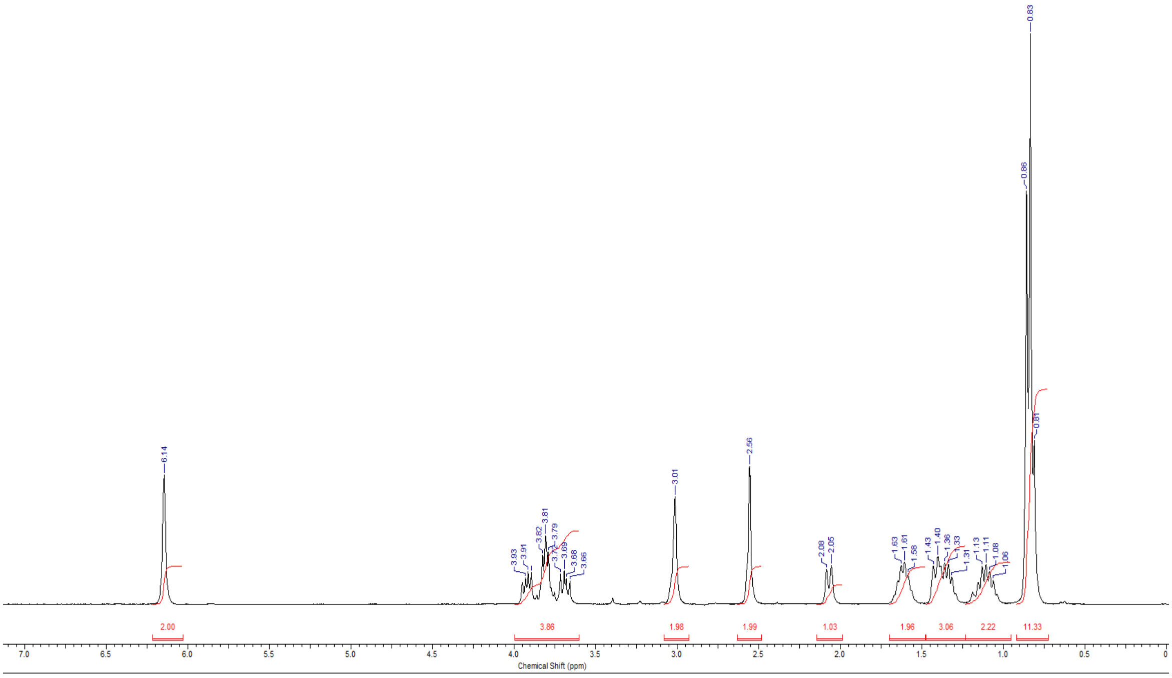

Figure S9. ^{1}H NMR spectrum of **M5** ($CDCl_3$).

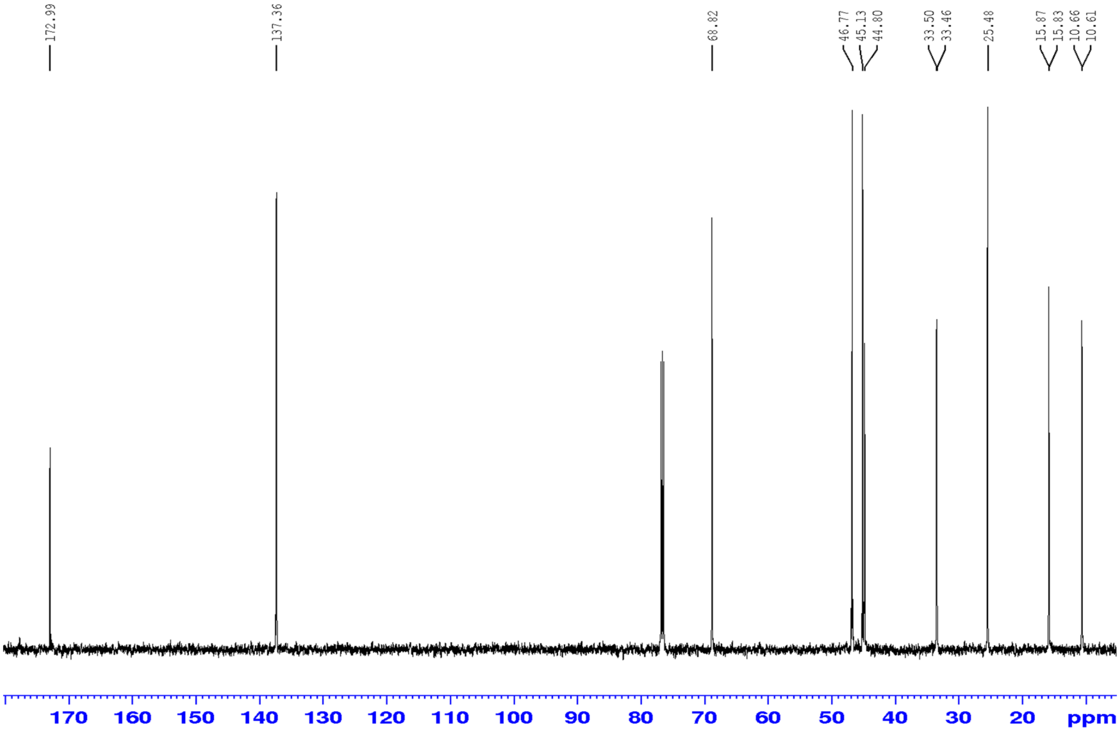

Figure S10. ^{13}C NMR spectrum of **M5** ($CDCl_3$).

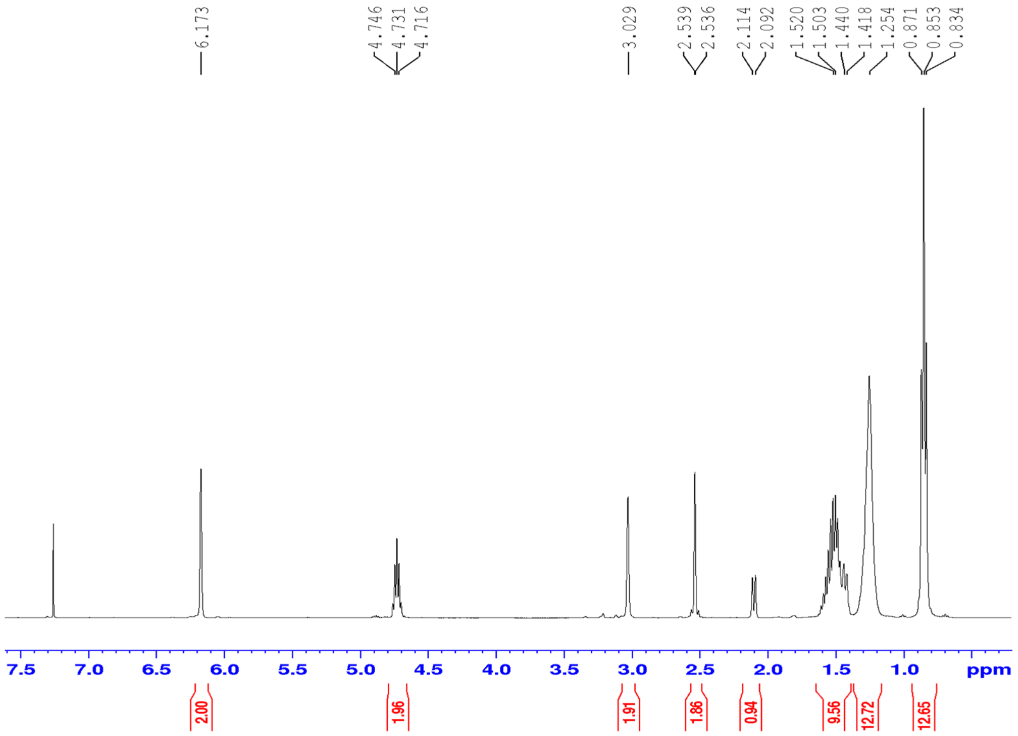

(a)

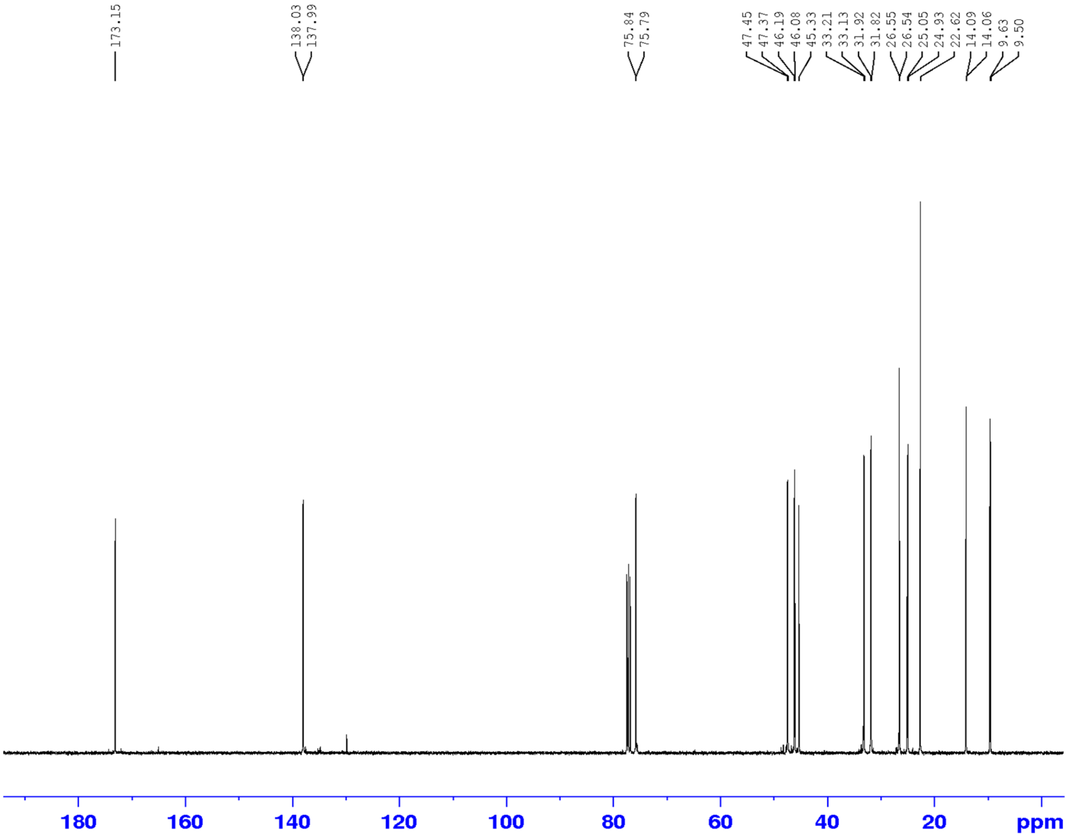

(b)

Figure S11. ^{1}H (a) and ^{13}C (b) NMR spectra of **M6** ($CDCl_3$).

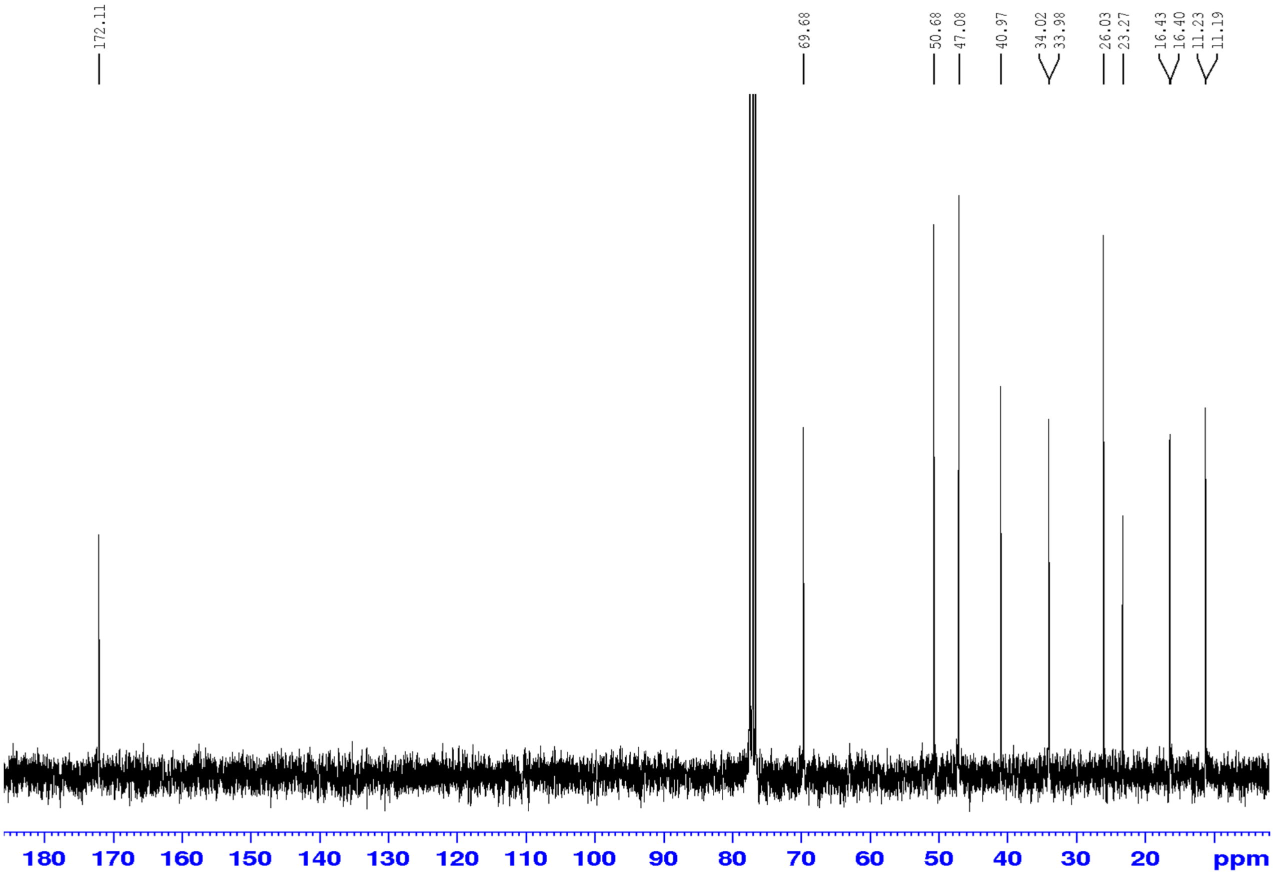

Figure S12. ^{13}C NMR spectrum of epoxide based on **M5** ($CDCl_3$).

ESI-HRMS spectra of the monomers

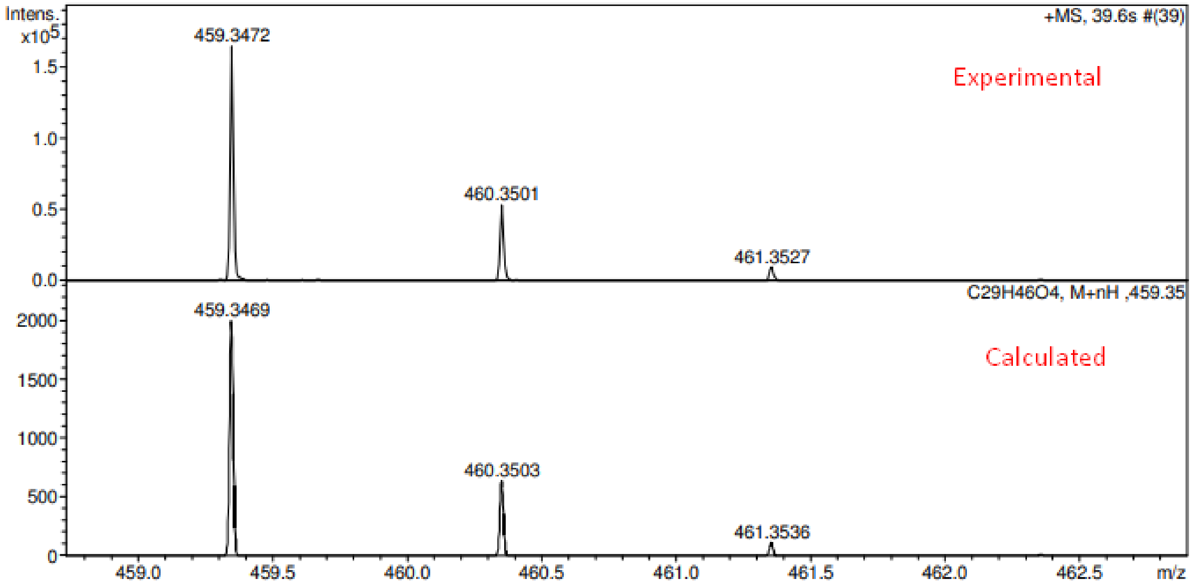

Figure S13. Experimental and theoretical ESI-(+)HRMS spectra of **M1** in CH_3CN solution: experimental peak $[M+H]^+$ = 459.3472 Da, calculated for $C_{29}H_{47}O_4$ = 459.3469.

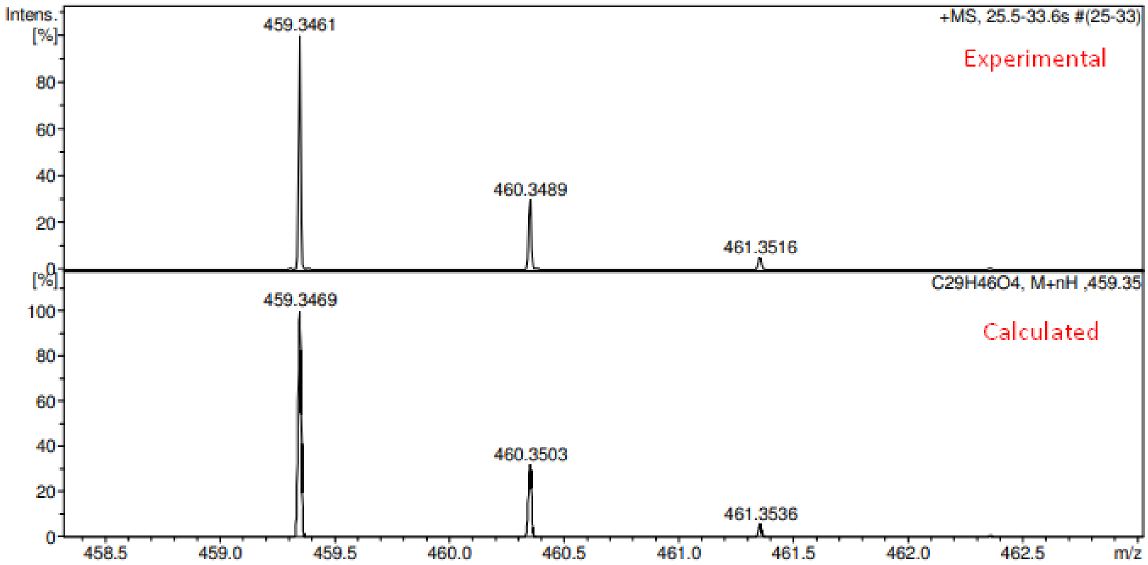

Figure S14. Experimental and theoretical ESI-(+)HRMS spectra of **M2** in CH_3CN solution: experimental peak $[M+H]^+$ = 459.3461 Da, calculated for $C_{29}H_{47}O_4$ = 459.3469.

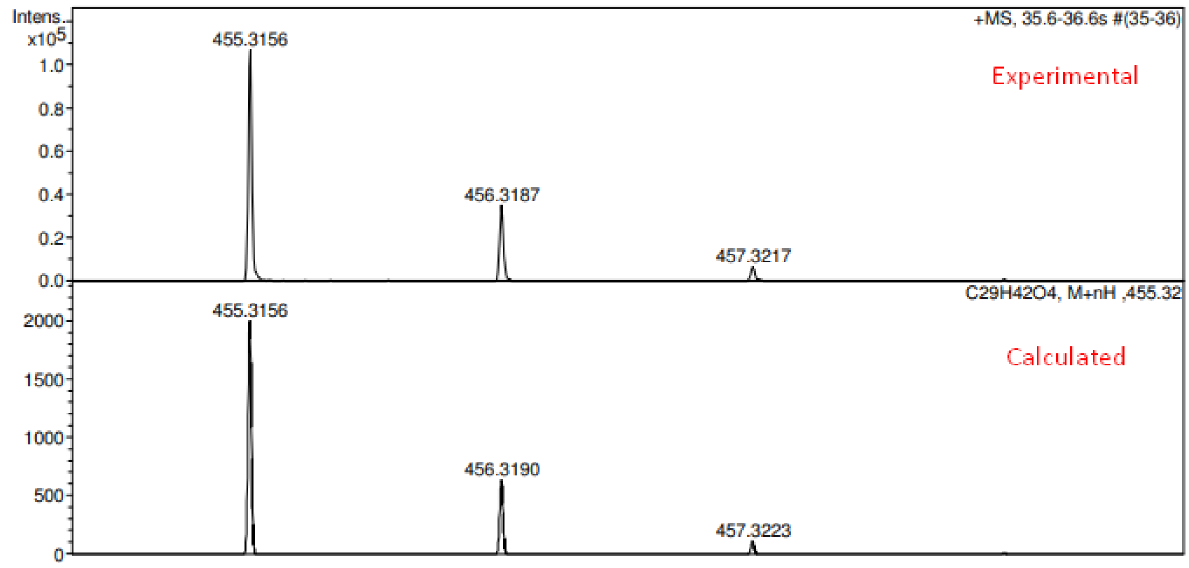

Figure S15. Experimental and theoretical ESI-(+)HRMS spectra of **M3** in CH_3CN solution: experimental peak $[M+H]^+$ = 455.3156 Da, calculated for $C_{29}H_{43}O_4$ = 455.3156

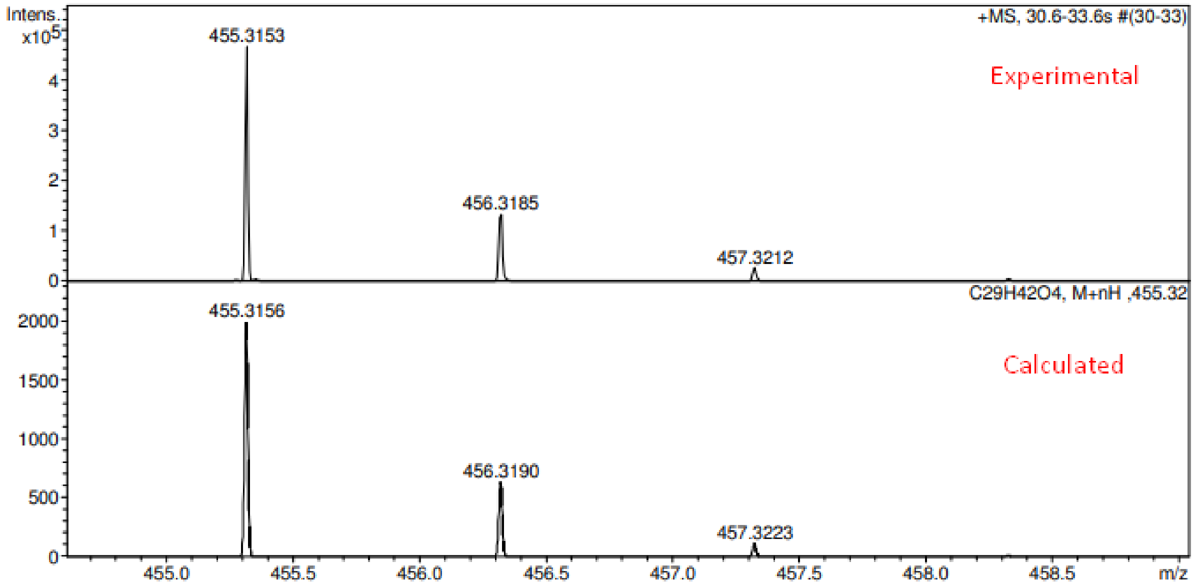

Figure S16. Experimental and theoretical ESI-(+)HRMS spectra of **M4** in CH_3CN solution: experimental peak $[M+H]^+$ = 455.3153 Da, calculated for $C_{29}H_{43}O_4$ = 455.3156.

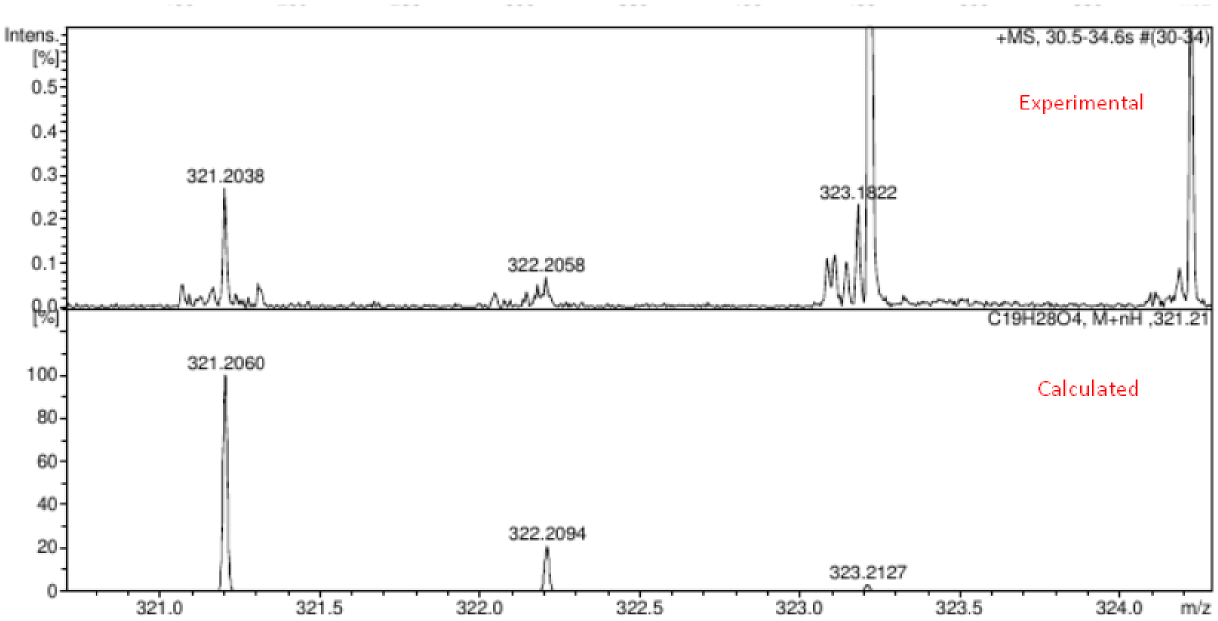

Figure S17. Experimental and theoretical ESI-(+)HRMS spectra of **M5** in CH_3CN solution: experimental peak $[M+H]^+$ = 321.2038 Da, calculated for $C_{29}H_{29}O_4$ = 321.2060 (MS is dirty due to some part of the monomer was converted to the epoxide).

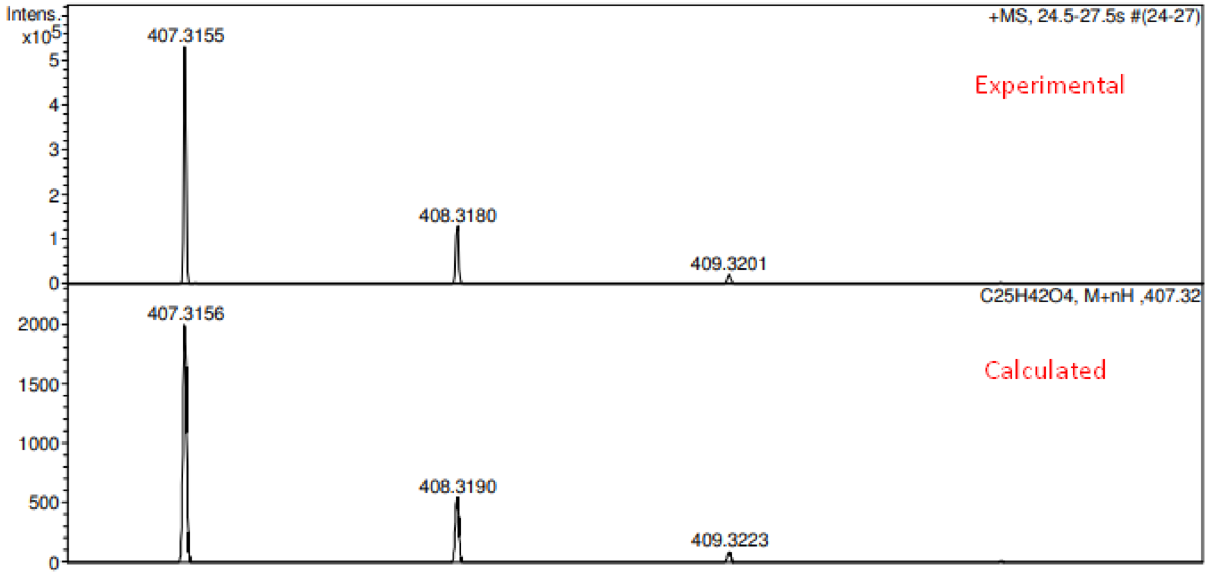

Figure S18. Experimental and theoretical ESI-(+)HRMS spectra of **M6** in CH_3CN solution: experimental peak $[M+H]^+$ = 407.3155 Da, calculated for $C_{25}H_{43}O_4$ = 407.3156.

X-ray diffraction data for M1 and M2

Table S1. Crystal data and structure refinement details for **M1** and **M2**.

Parameter	**M1**	**M2**
Formula	$C_{29}H_{46}O_4$	$C_{29}H_{46}O_4$
M	458.66	458.66
T, K	100	120
Crystal system	Orthorhombic	Monoclinic
Space group	$P2_12_12_1$	$C2$
Z (Z')	4 (1)	4 (1)
a, Å	6.0929(10)	23.4647(18)
b, Å	36.257(3)	5.7765(4)
c, Å	12.0966(14)	21.9950(17)
α, deg	90	90
β, deg	90	115.155(4)
γ, deg	90	90
V, Å^3	2672.3(6)	2698.5(4)
d_{calcd}, g·cm^{-3}	1.140	1.129
μ, mm^{-1}	0.74	5.71
F_{000}	1008	1008
Θ_{max}, deg	50	130
Number of measured refl.	22808	12573
Number of independent refl.	4665	4051
Observed refl. [$I > 2\sigma(I)$]	3526	3131
Parameters	305	306
R_1 [$F^2 > 2\sigma(F^2)$]	0.0926	0.0499
wR_2 (all data)	0.2202	0.1461
$S(F^2)$	1.003	1.069
Residual electron density (d_{max}/d_{min}), e·Å^{-3}	0.371/-0.291	0.289/-0.256

NMR spectra of the ROMP polymers

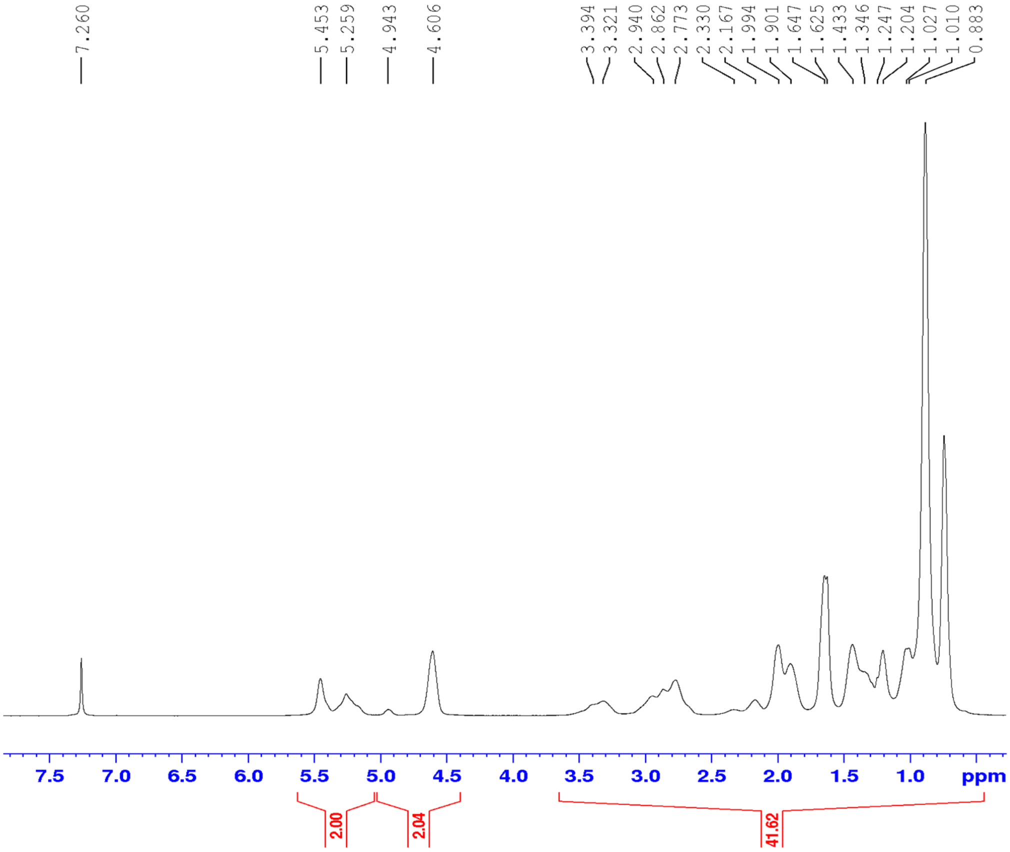

Figure S19. ^{1}H NMR spectrum of **MP1** ($CDCl_3$).

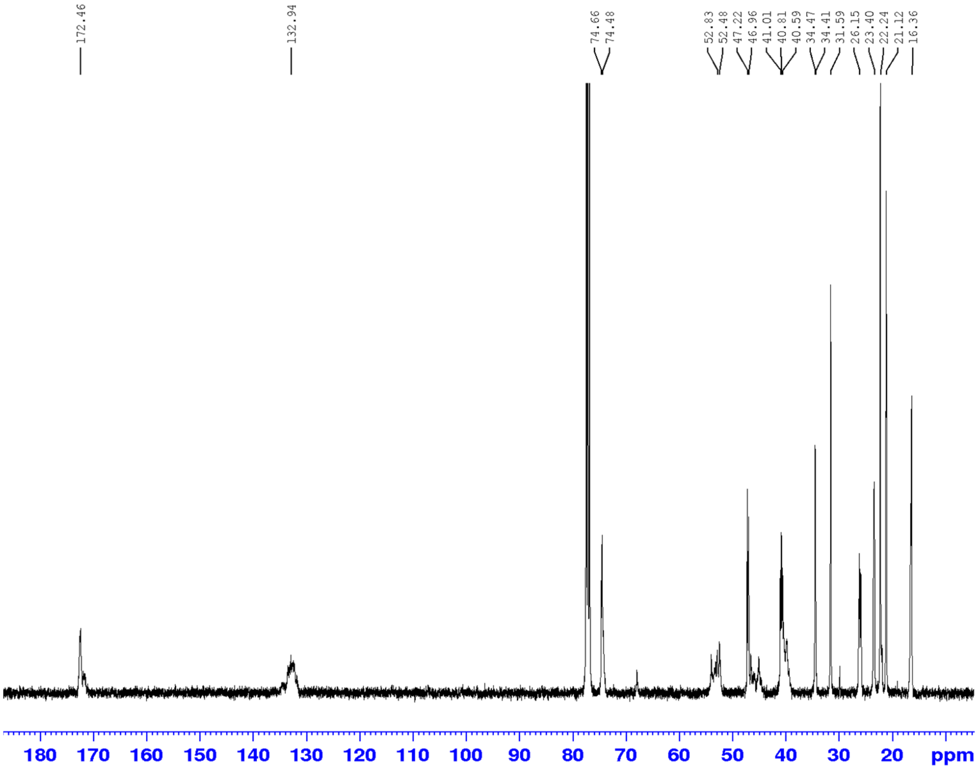

Figure S20. ^{13}C NMR spectrum of **MP1** ($CDCl_3$).

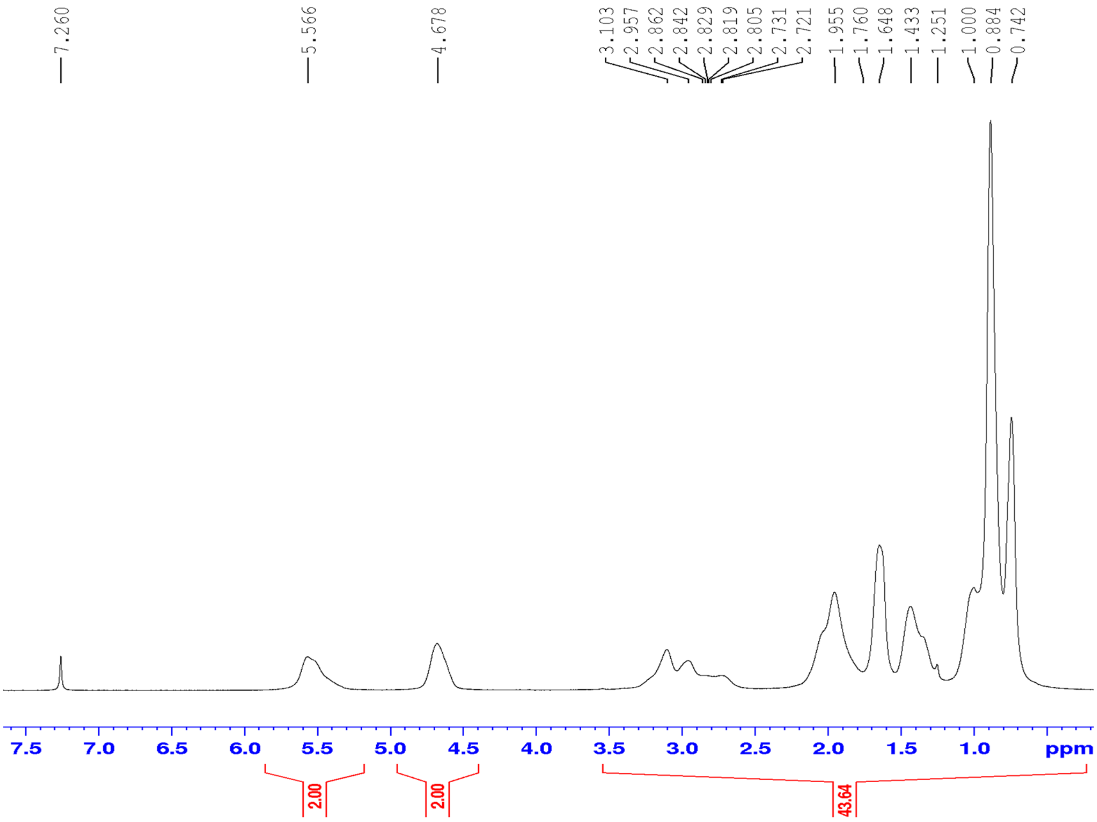

Figure S21. ^{1}H NMR spectrum of **MP2** ($CDCl_3$).

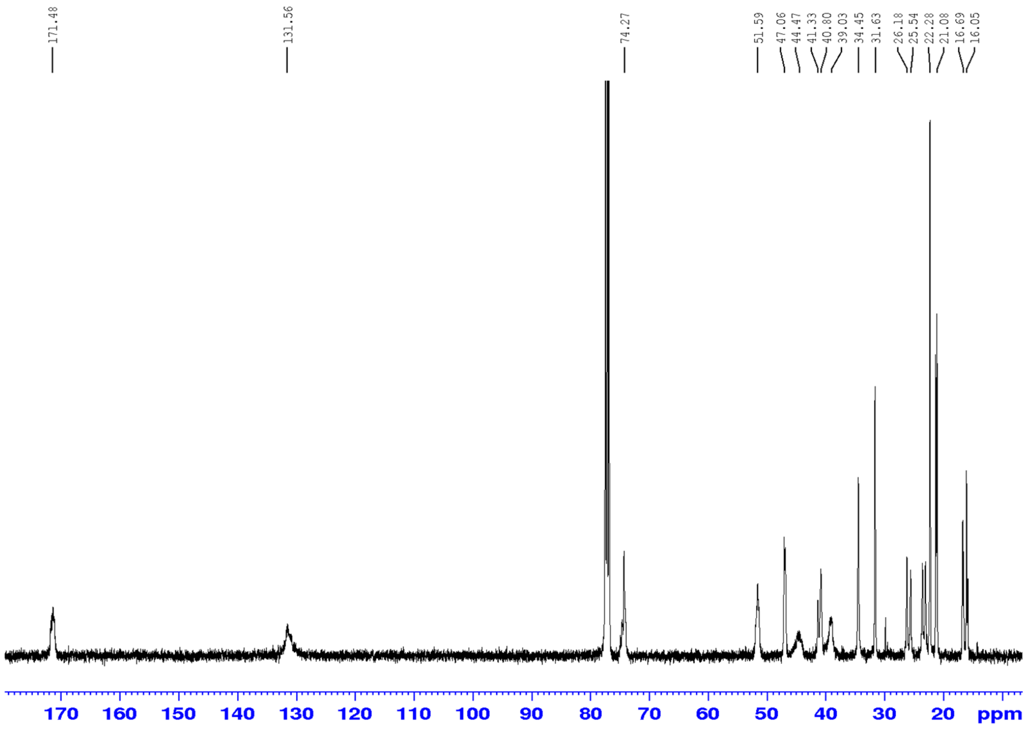

Figure S22. ^{13}C NMR spectrum of **MP2** ($CDCl_3$).

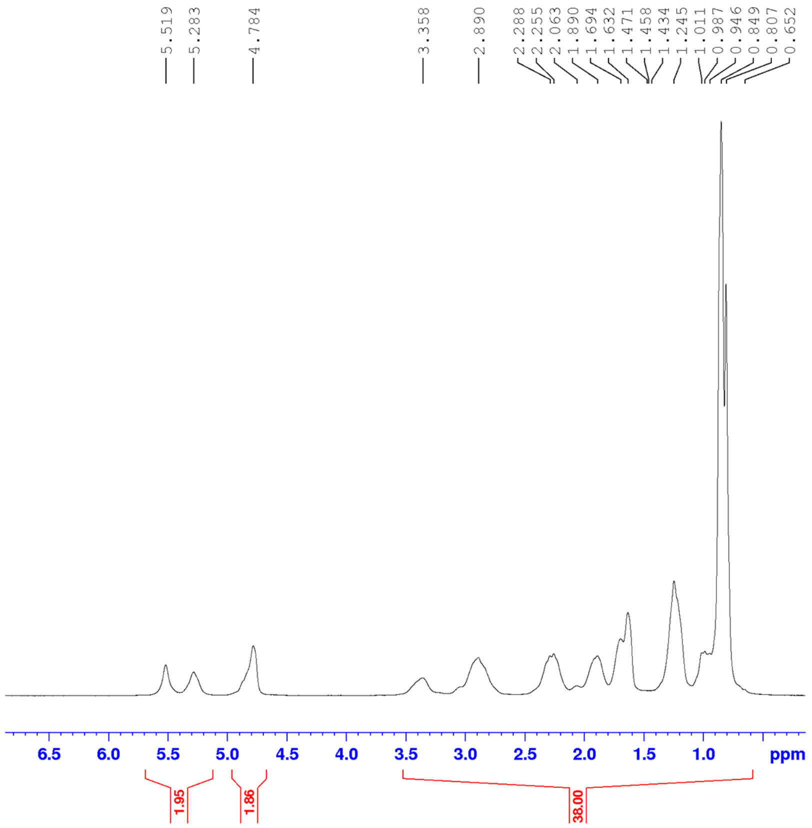

Figure S23. ^{1}H NMR spectrum of **MP3** ($CDCl_3$).

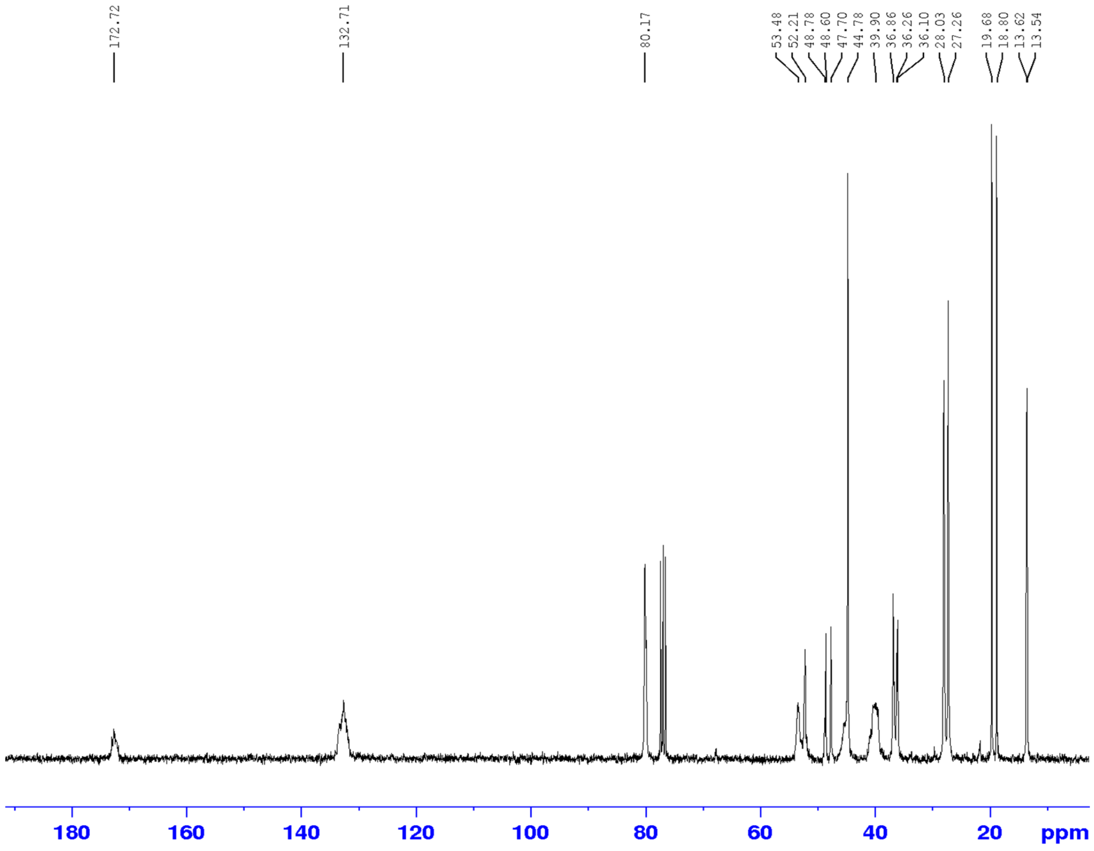

Figure S24. ^{13}C NMR spectrum of **MP3** ($CDCl_3$).

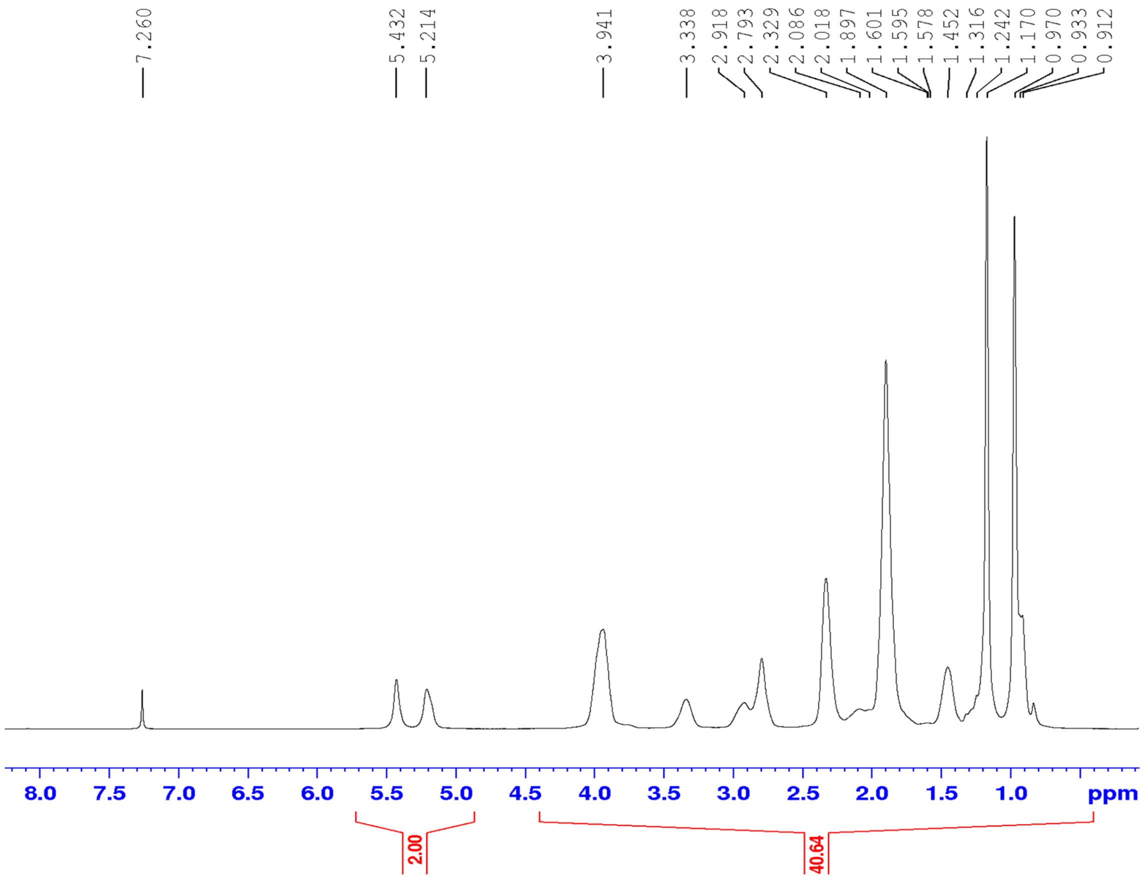

Figure S25. ^{1}H NMR spectrum of **MP4** ($CDCl_3$).

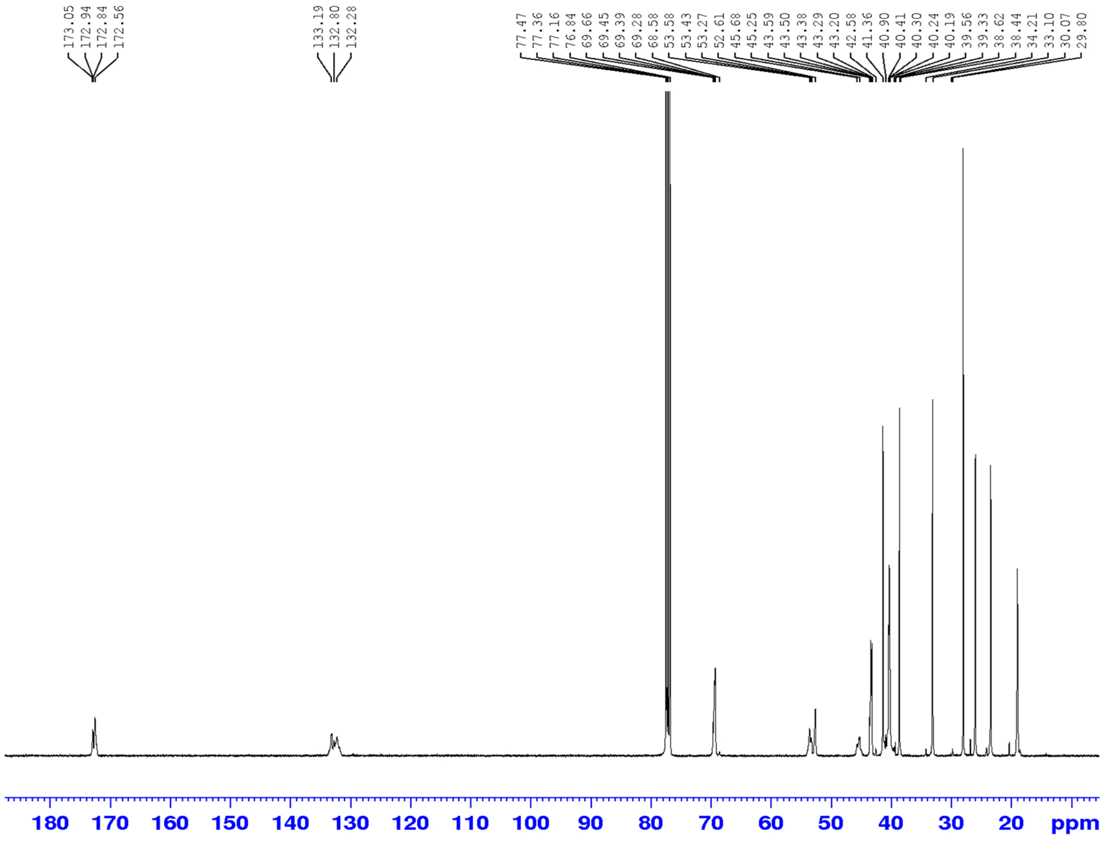

Figure S26. ^{13}C NMR spectrum of **MP4** ($CDCl_3$).

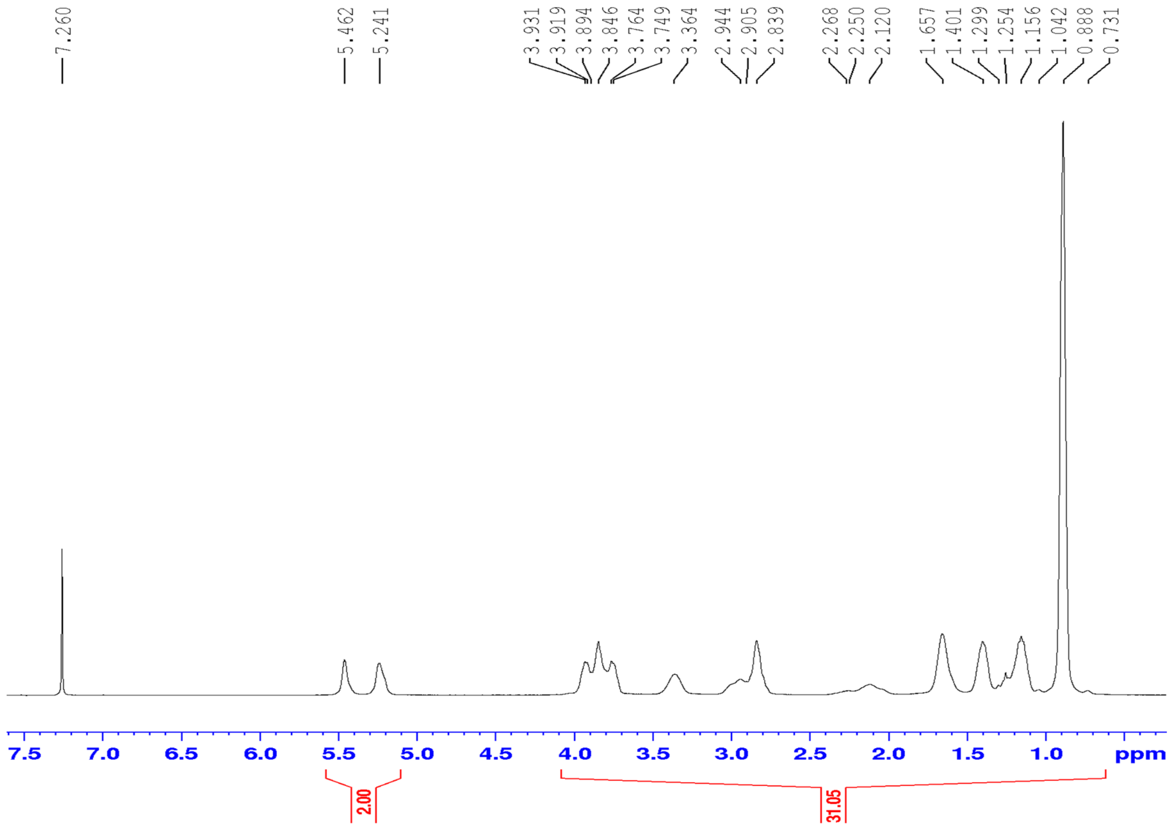

Figure S27. ^{1}H NMR spectrum of **MP5** ($CDCl_3$).

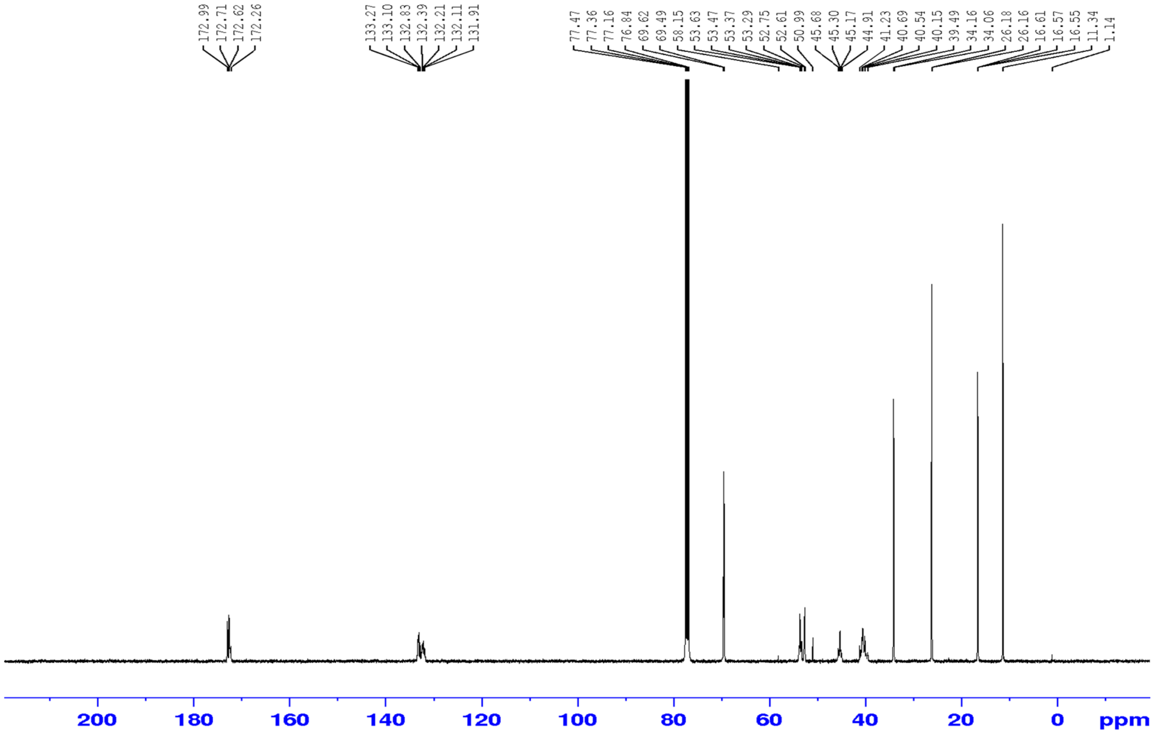

Figure S28. ^{13}C NMR spectrum of **MP5** ($CDCl_3$).

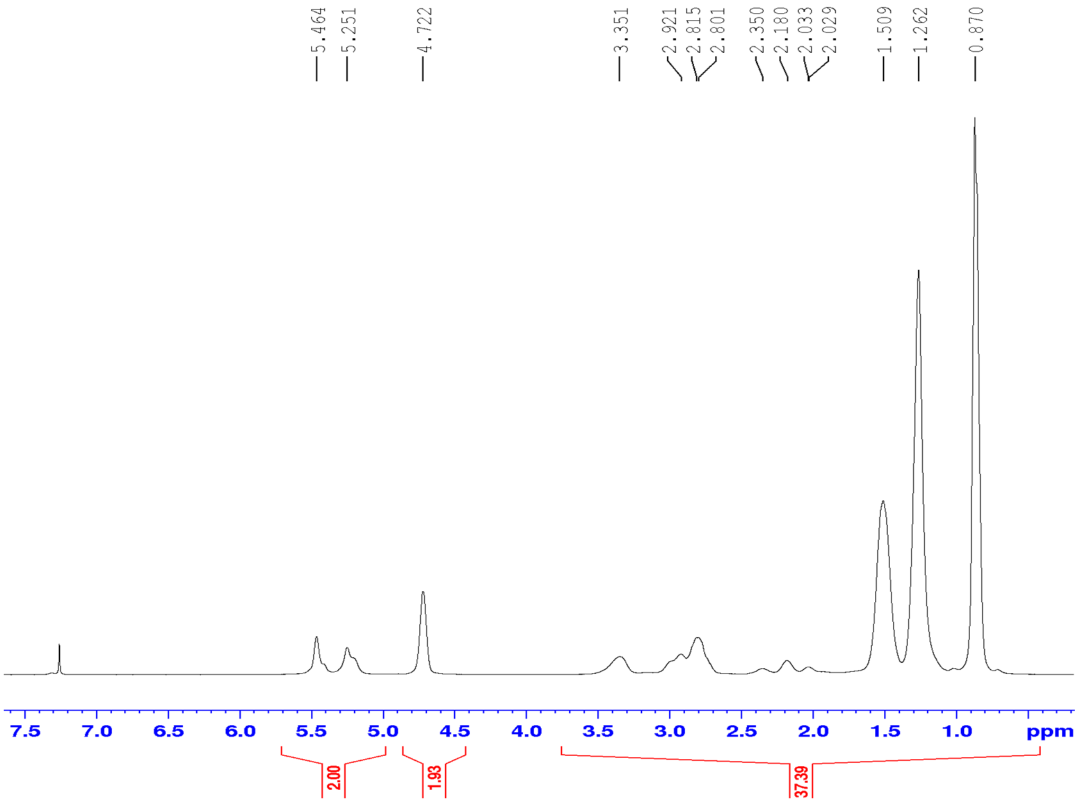

(a)

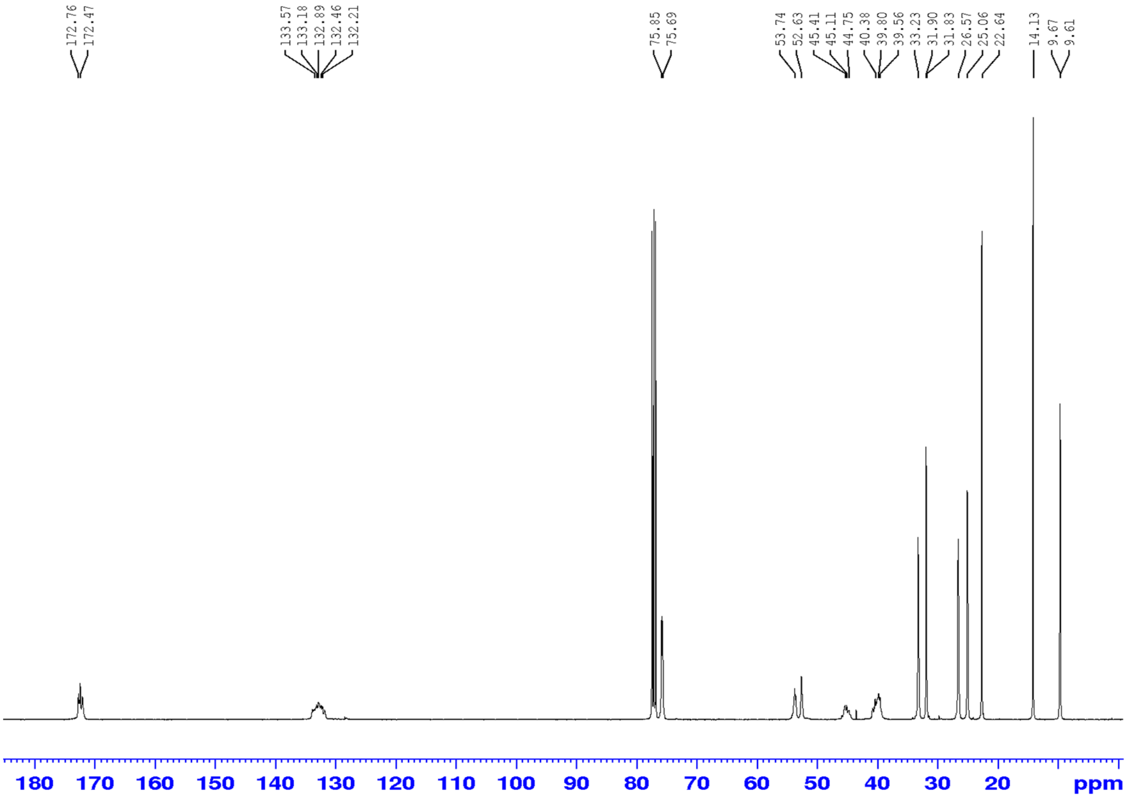

(b)

Figure S29. (a) ^{1}H and (b) ^{13}C NMR spectra of **MP6** ($CDCl_3$).

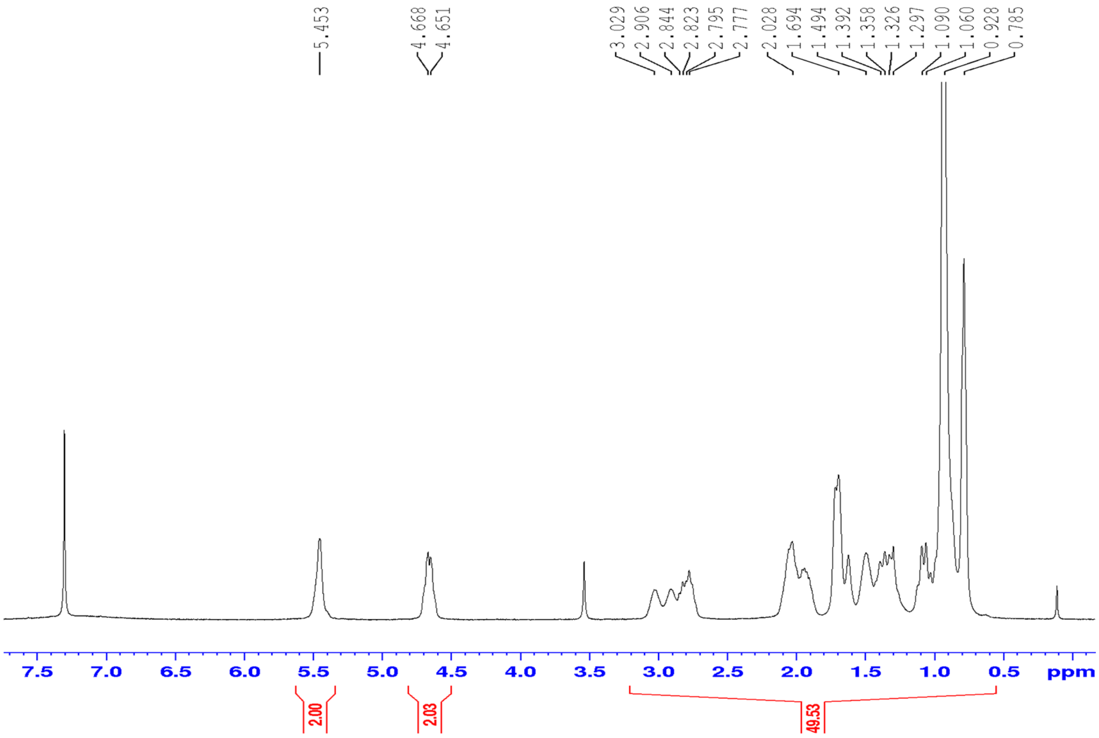

Figure S30. ^{1}H NMR spectrum of **MP7** ($CDCl_3$).

CD spectra of the monomers and the ROMP polymers

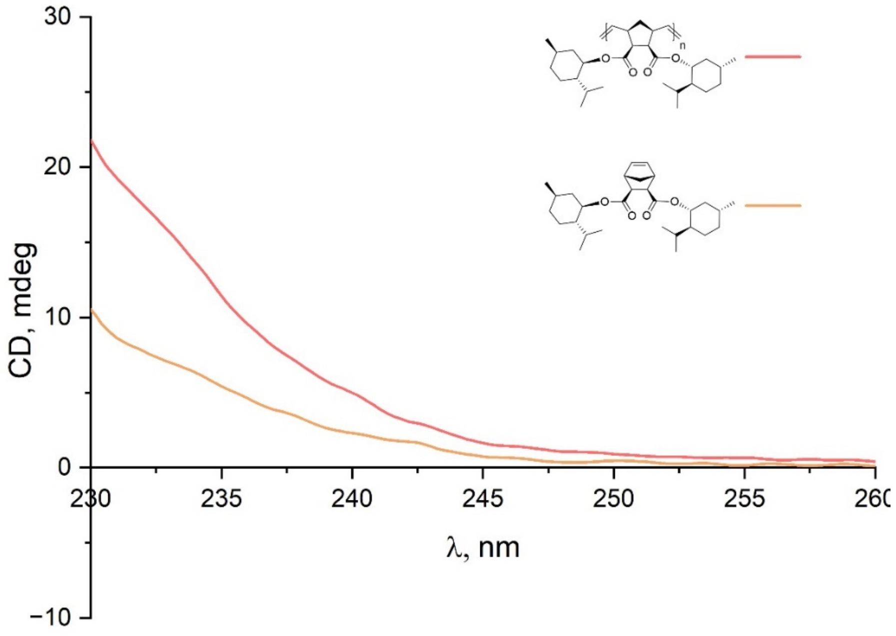

Figure S31. CD spectra of **M1** and **MP1**.

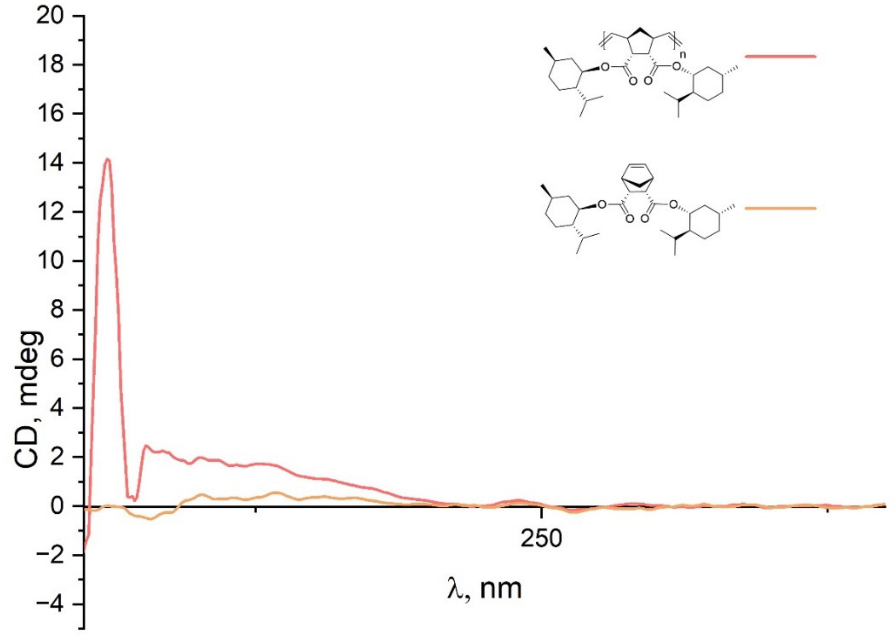

Figure S32. CD spectra of **M2** and **MP2**.

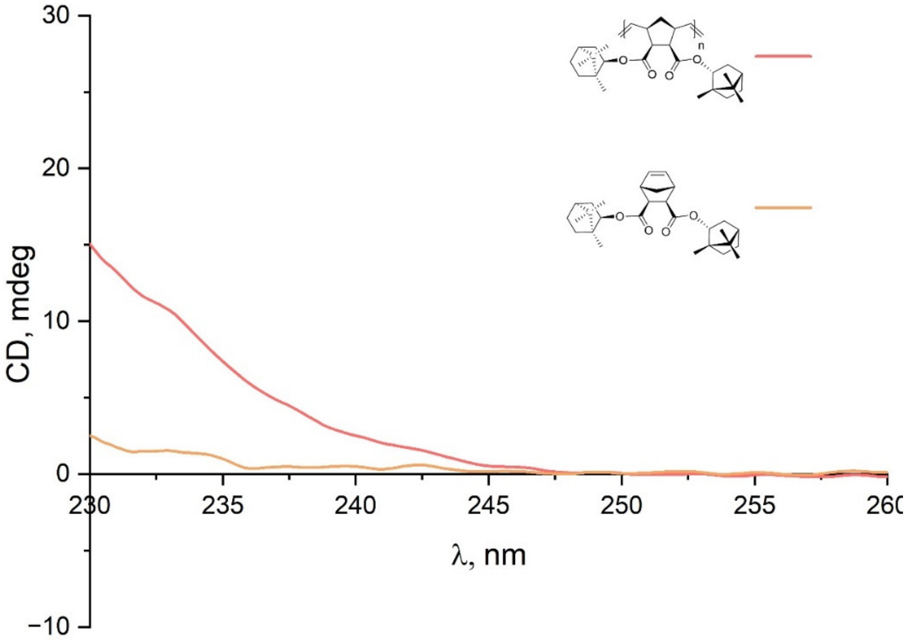

Figure S33. CD spectra of **M3** and **MP3**.

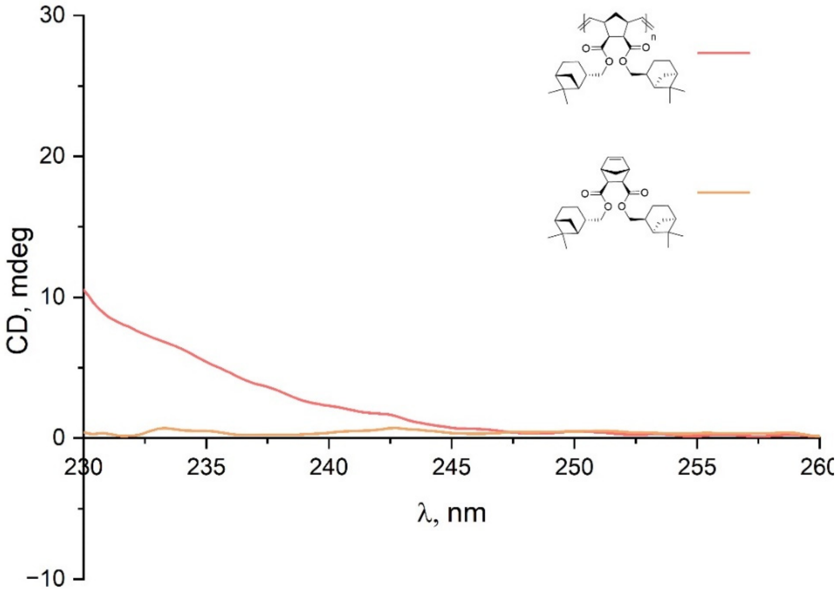

Figure S34. CD spectra of **M4** and **MP4**.

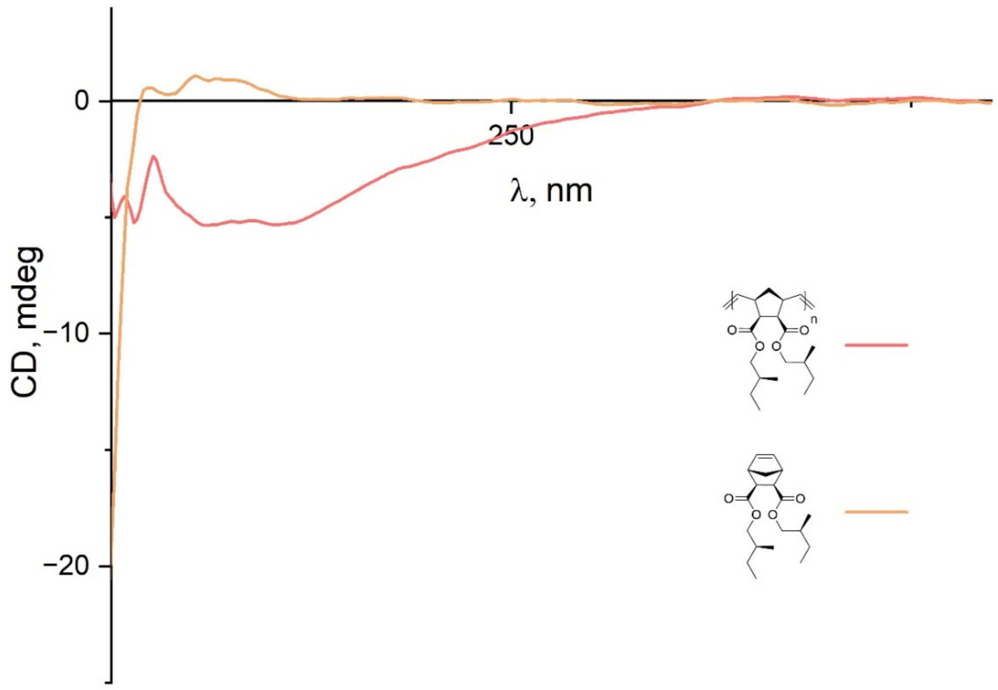

(a)

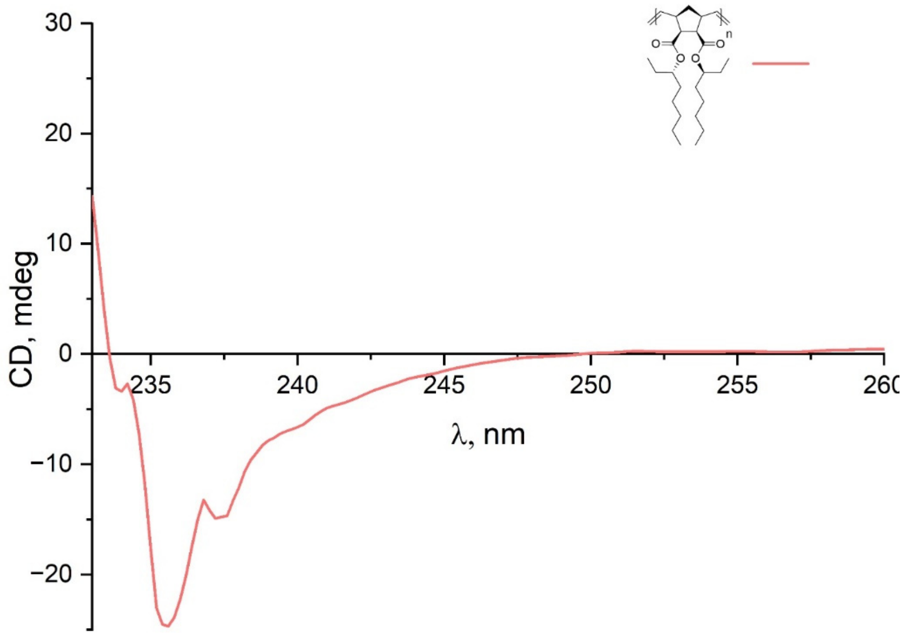

(b)

Figure S35. CD spectra of (a) **M5** and **MP5**, and (b) **MP6**.

DSC curves of the polymers

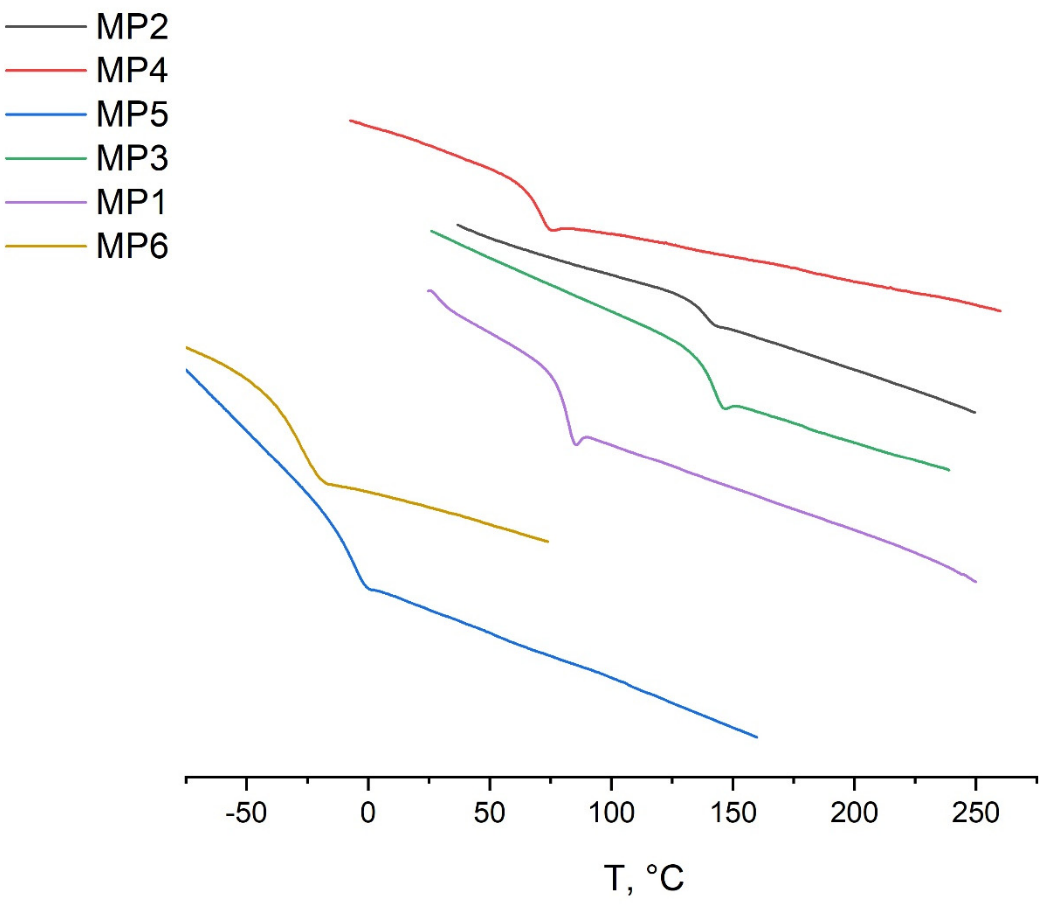

Figure S36. DSC curves of the prepared ROMP polymers.

TGA curves of the ROMP polymers

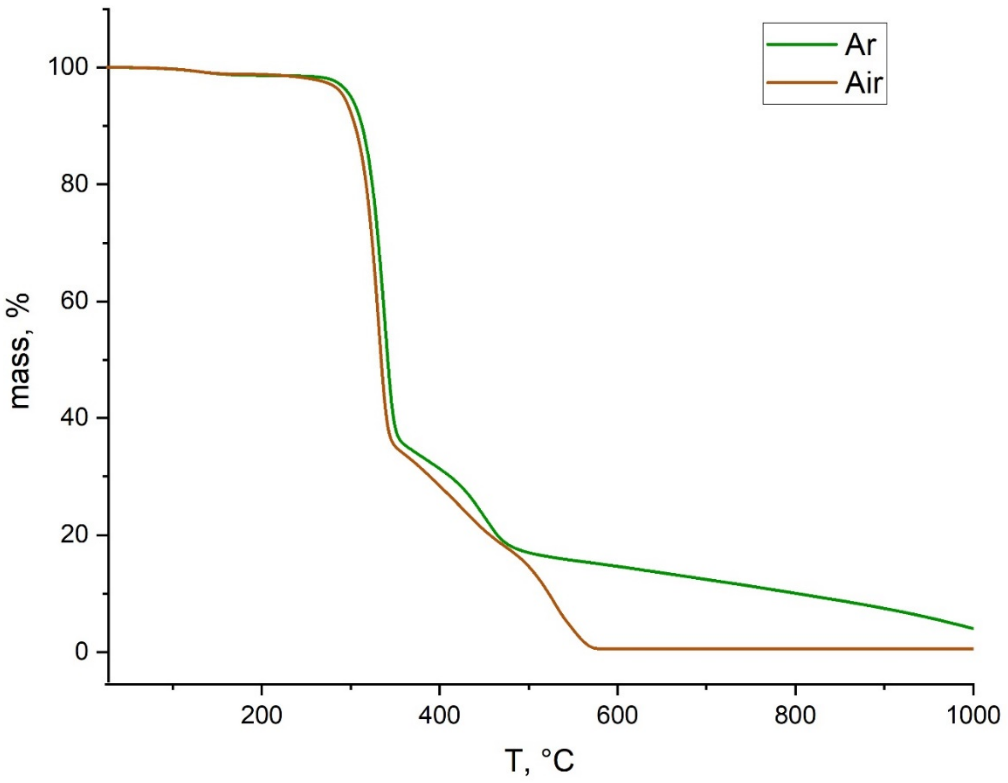

Figure S37. TGA curves for **MP1.**

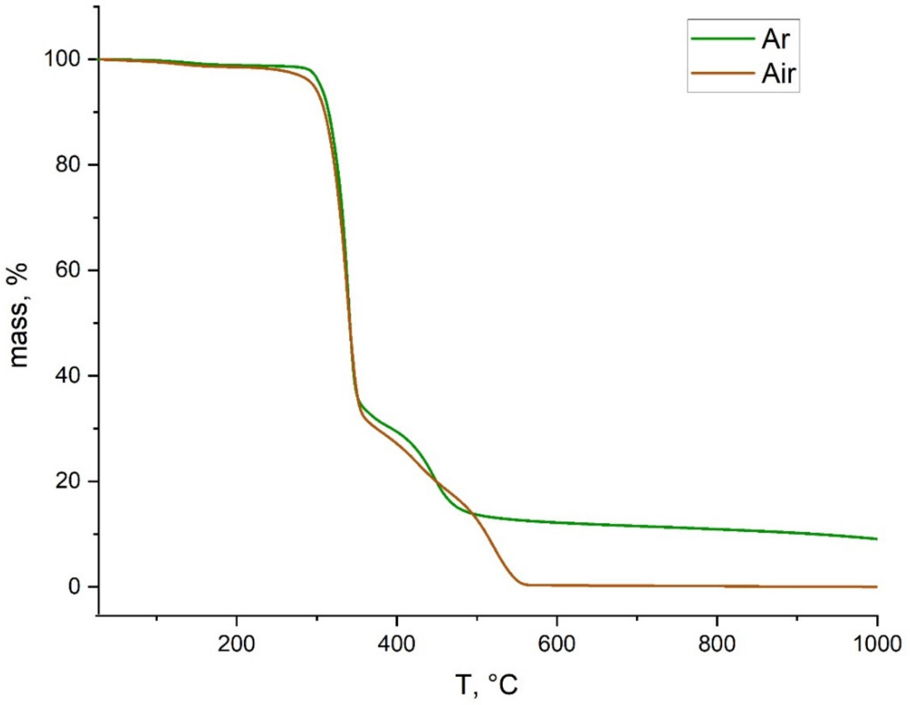

Figure S38. TGA curves for **MP2.**

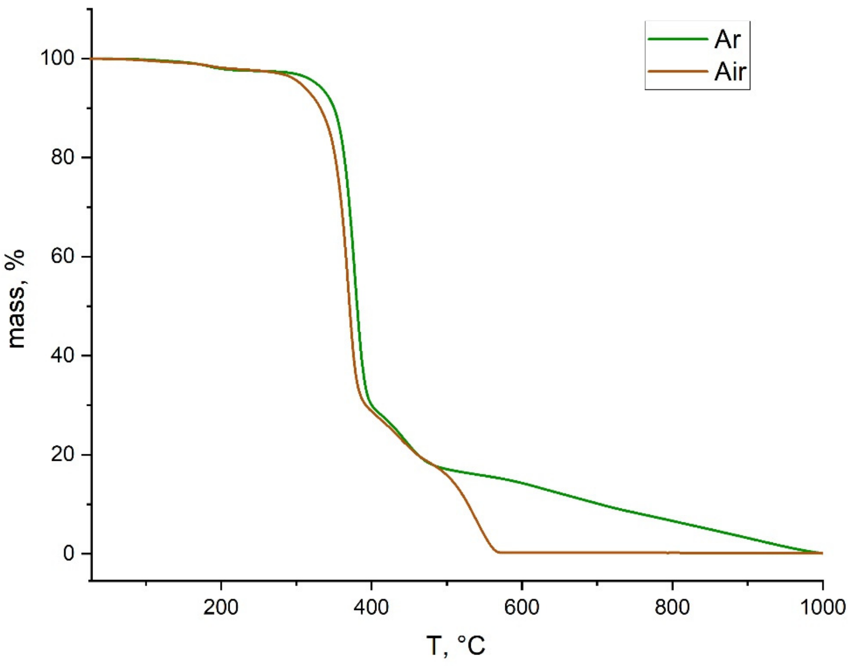

Figure S39. TGA curves for **MP3**.

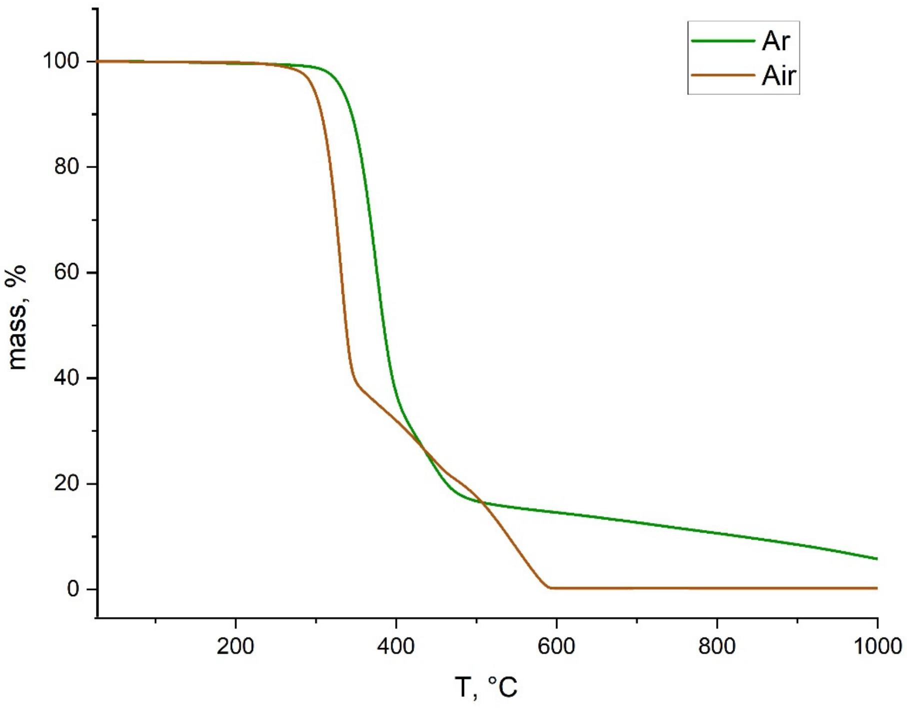

Figure S40. TGA curves for **MP4**.

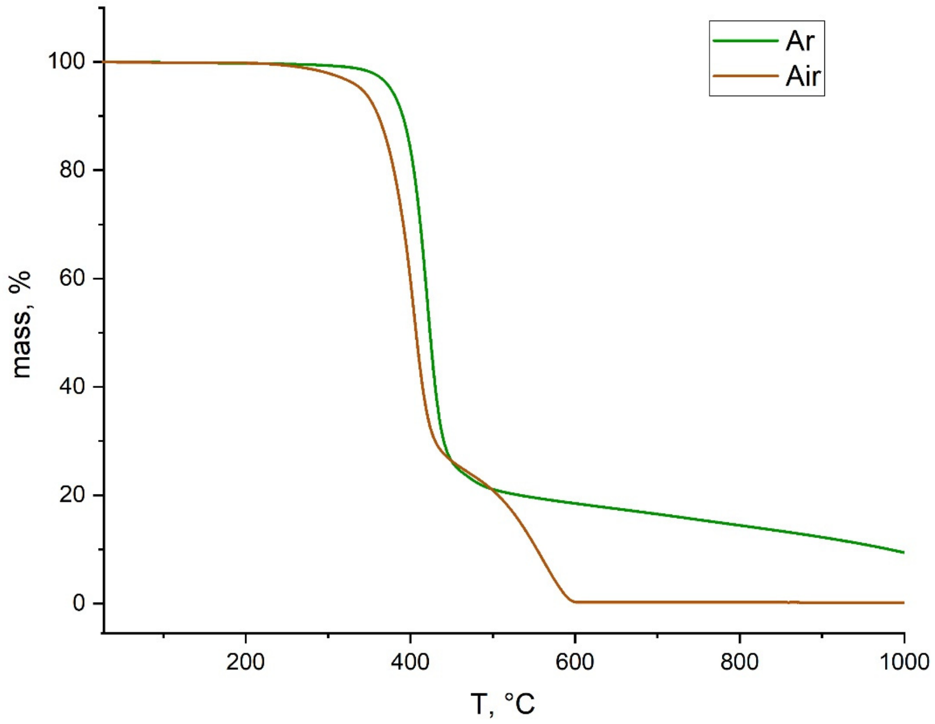

Figure S41. TGA curves for **MP5**.

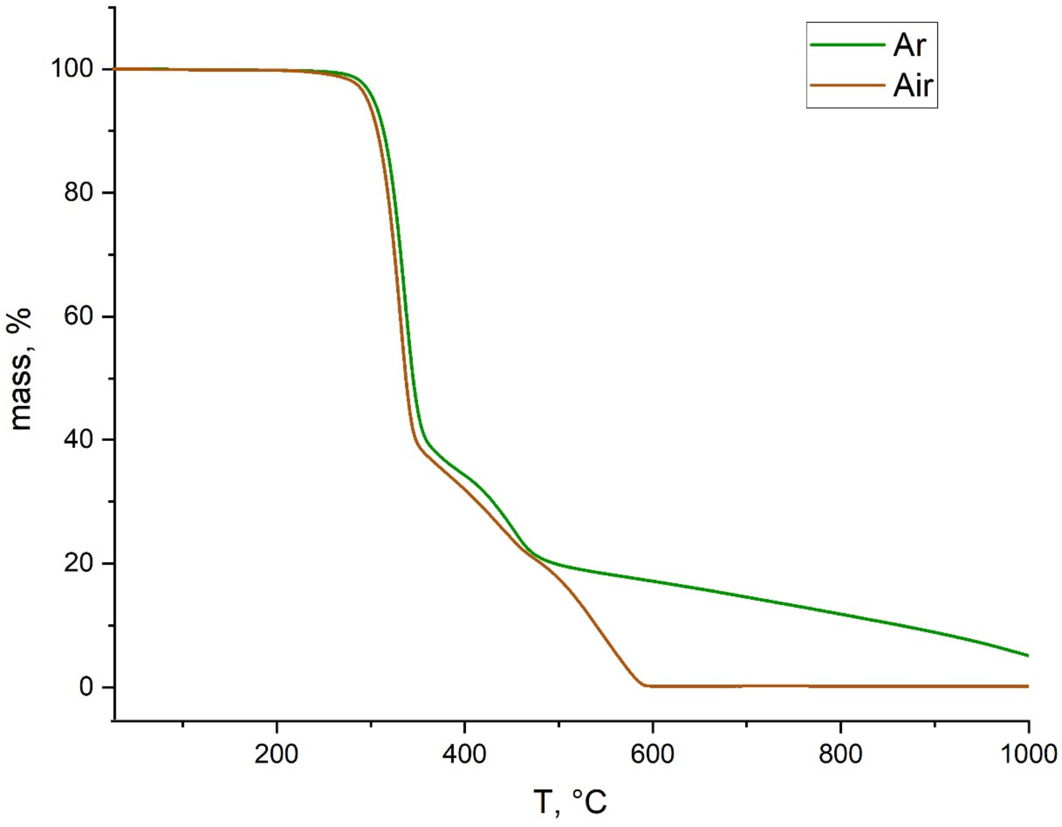

Figure S42. TGA curves for **MP6**.

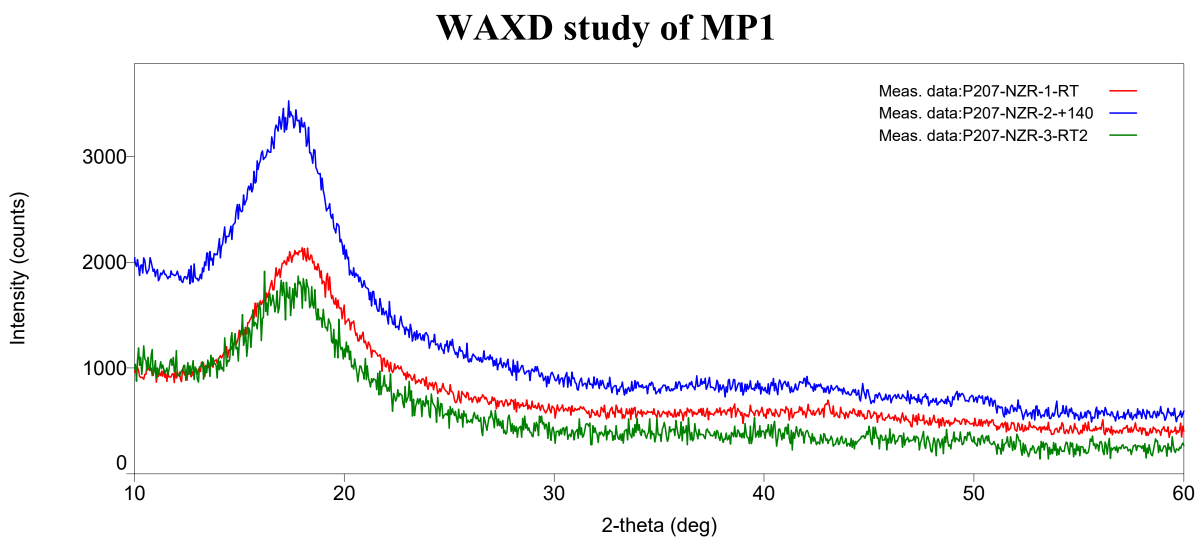

Figure S43. WAXD patterns of MP1 before (the middle red line, obtained at room temperature), after heating at 140ºC (the bottom green line, obtained at room temperature), and at 140ºC (the top blue line).
