# Supplementary figures and images for: Chiral Polymers from Norbornenes Based on Renewable Chemical Feedstocks
Source: Polymers (Basel). 2022 Dec 13;14(24):5453. doi: 10.3390/polym14245453 (PMC9786787; doi:10.3390/polym14245453)

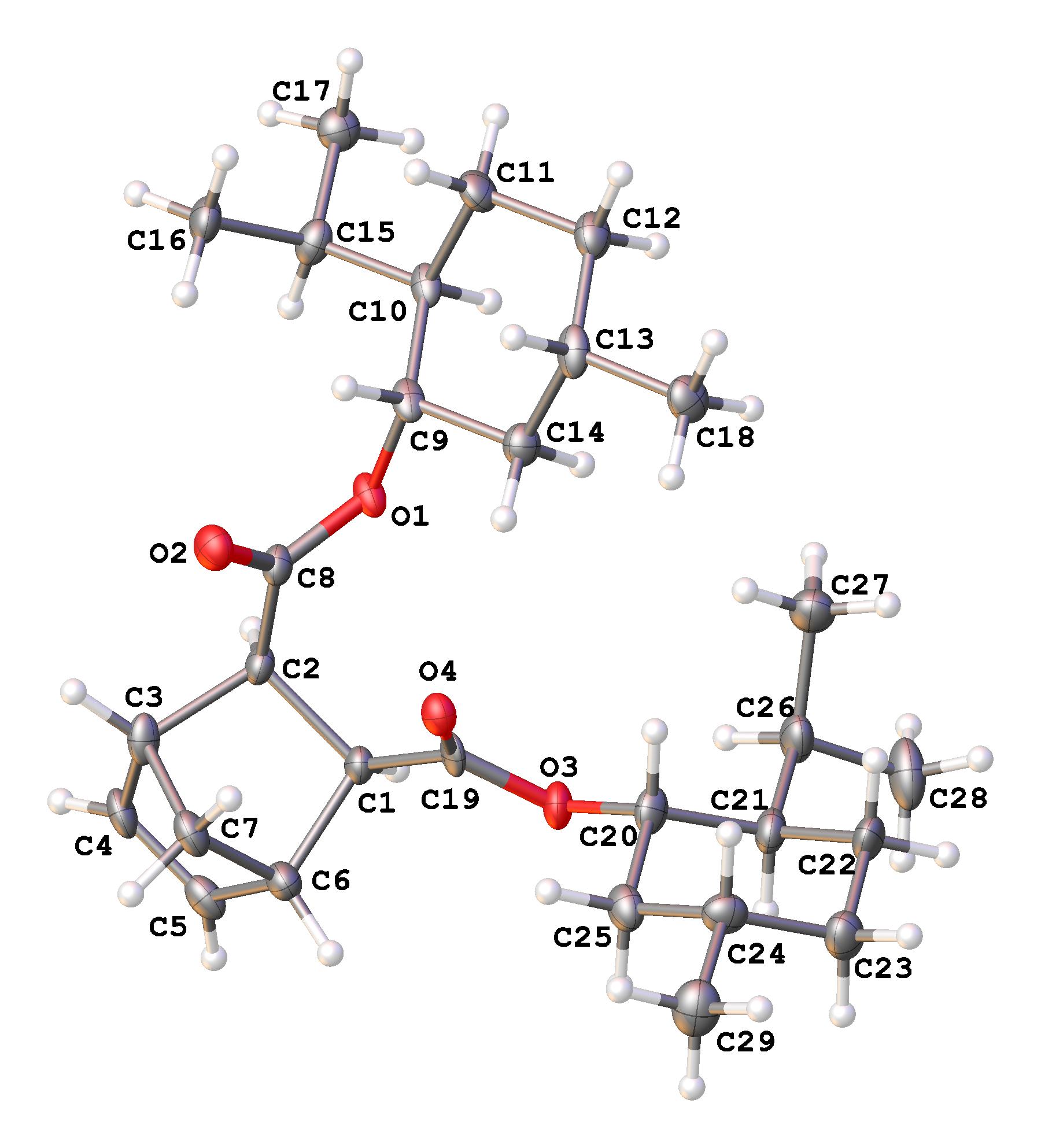

Supplement: Supplementary file 1 [file polymers-14-05453-s001.zip › NZR156_H_telp50.tif]

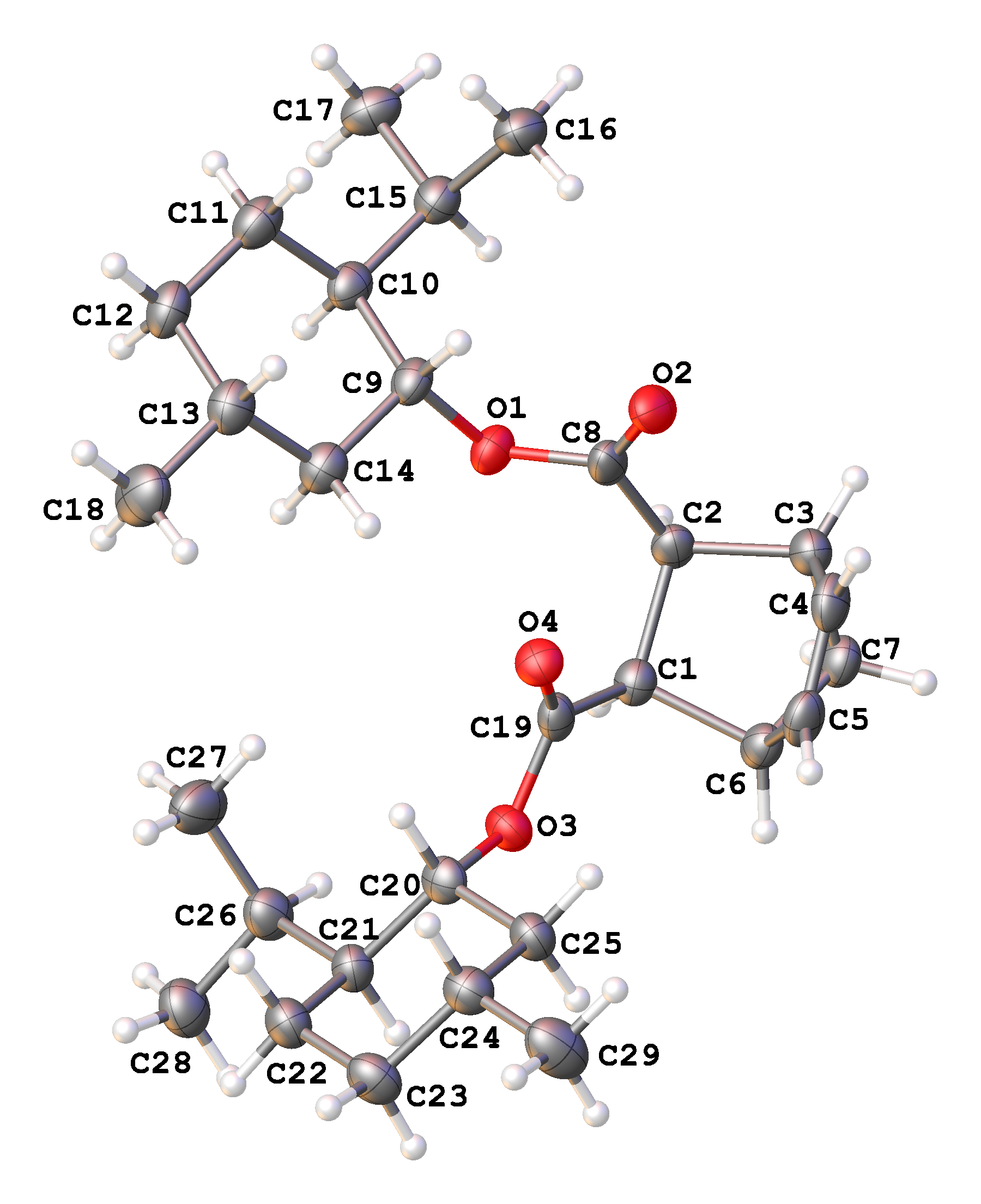

Supplement: Supplementary file 1 [file polymers-14-05453-s001.zip › NZR3711_h_var1_telp50.tif]
